# Cut from the Same Cloth: Enamine-Derived Spirobifluorenes as Hole Transporters for Perovskite Solar Cells

**DOI:** 10.1021/acs.chemmater.1c01486

**Published:** 2021-07-19

**Authors:** Deimante Vaitukaityte, Cristina Momblona, Kasparas Rakstys, Albertus Adrian Sutanto, Bin Ding, Cansu Igci, Vygintas Jankauskas, Alytis Gruodis, Tadas Malinauskas, Abdullah M. Asiri, Paul J. Dyson, Vytautas Getautis, Mohammad Khaja Nazeeruddin

**Affiliations:** † Department of Organic Chemistry, Kaunas University of Technology, Radvilenu pl. 19, Kaunas 50254, Lithuania; ‡ Institute of Chemical Sciences and Engineering, École Polytechnique Fédérale de Lausanne, CH-1951 Sion, Switzerland; § Institute of Chemical Physics, Vilnius University, Sauletekio al. 3, Vilnius 10257, Lithuania; ∥ Center of Excellence for Advanced Materials Research (CEAMR), King Abdulaziz University, P.O. Box 80203, 21589 Jeddah, Saudi Arabia

## Abstract

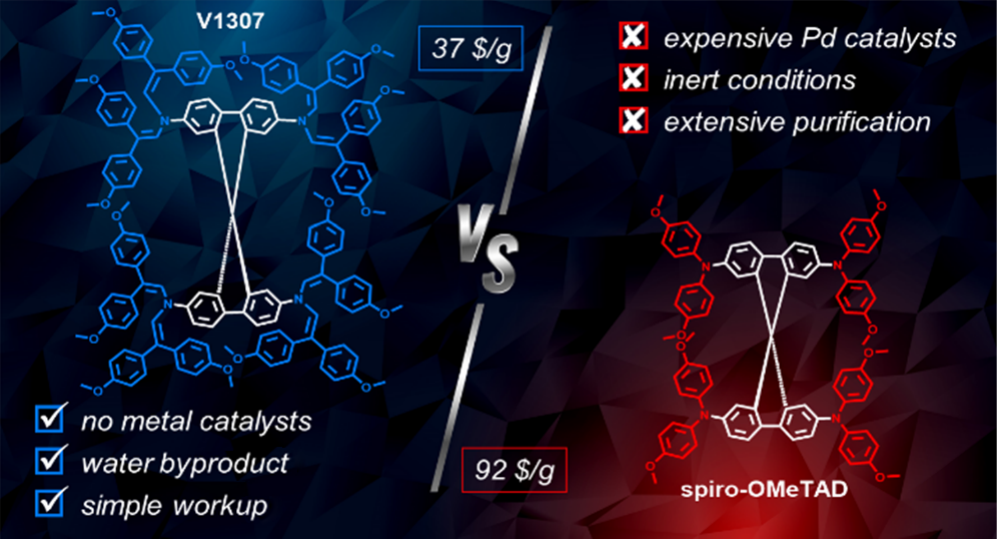

To attain commercial viability, perovskite solar cells (PSCs) have to be reasonably priced, highly efficient, and stable for a long period of time. Although a new record of a certified power conversion efficiency (PCE) value over 25% was achieved, PSC performance is limited by the lack of hole-transporting materials (HTMs), which extract positive charges from the light-absorbing perovskite layer and carry them to the electrode. Here, we report spirobifluorene-based HTMs with finely tuned energy levels, high glass-transition temperature, and excellent charge mobility and conductivity enabled by molecularly engineered enamine arms. HTMs are synthesized using simple condensation chemistry, which does not require costly catalysts, inert reaction conditions, and time-consuming product purification procedures. Enamine-derived HTMs allow the fabrication of PSCs reaching a maximum PCE of 19.2% and stability comparable to spiro-OMeTAD. This work demonstrates that simple enamine condensation reactions could be used as a universal path to obtain HTMs for highly efficient and stable PSCs.

## Introduction

The perovskite materials used in solar cells, i.e., perovskite solar cells (PSCs), and *A*Pb*X*_3_ (*A* = methylammonium (MA), formamidinium (FA), caesium (Cs); *X* = Br, I) have remarkable properties, such as high absorption coefficients, long carrier diffusion lengths, small exciton binding energies, and high charge carrier mobilities.^1−7^ Most highly efficient PSCs utilize an n-type layer of mesoporous TiO_2_ and a p-type layer of spiro-OMeTAD in an n–i–p device configuration, where perovskite materials are used as light absorbers.^8^ With the increased quality of perovskite films, further optimization of other layers to improve the overall solar cell performance is needed.^9,10^ Specifically, there is a renewed interest in identifying hole-transporting materials (HTMs) other than spiro-OMeTAD that can yield high power conversion efficiency (PCE).

Since the first report of an HTM in solid-state dye-sensitized solar cells 2 decades ago, the organic semiconductor 2,2′,7,7′-tetrakis-(*N*,*N*′-di-*p*-methoxyphenylamine)-9,9′-spirobifluorene (spiro-OMeTAD) has revolutionized the field and has been selected as the state-of-the-art benchmark.^11^ Two decades have passed, yet spiro-OMeTAD still prevails among other HTMs in the field of PSCs, and despite its high cost (∼250 $/g), it is commonly used as a highly efficient reference material for research studies. The wide availability of spiro-OMeTAD due to commercialization decades ago makes it one of the most researched materials, which is currently reported more than 4000 times in the scientific literature. However, the cost-effective industrial potential of spiro-OMeTAD toward practical applications is hardly probable due to its synthetic complexity and high-purity sublimation-grade requirement to obtain high-performance devices contributing more than 30% of the overall module price. Another key factor that plays a major role in the commercialization potential is the stability of the device. To match the necessary electrical conductivity, spiro-OMeTAD needs to be doped as pristine layers generally suffer from low PCE.^12,13^

Due to the success of spiro-OMeTAD, many research groups have focused on spiro-type compounds, expecting to improve the PCE with slight structural modifications.^14−16^ The basic idea of the spiro concept is that the morphological stability is improved while retaining the electronic properties of connected π systems with identical or different functions via a common sp^3^-hybridized atom.^17^ Several groups have studied central 9,9′spirobifluorene-linked HTMs including dimethylfluorenyl-, ethylcarbazolyl-, and fluorinated methoxyphenyl-terminated compounds, which have been recently reported by Seo,^18^ Chen,^19^ and Yang,^20^ respectively. However, the main focus is still directed on the development of new central spiro-cored structures such as spiro[fluorene-9,9′-xanthene],^21−25^ spirobisacridine,^26^ thiophene-containing spiro cores,^27−29^ and other spiro-type derivatives.^30,31^ However, the peripheral part is equally or of even more importance in fine tuning the properties of the HTM.^32,33^ Generally, such spiro-centered HTMs are designed by linking prebrominated spiro core species with diphenylamine or borylated triphenylamine, both having electron-rich methoxy groups using C–N or C–C cross-coupling reactions, respectively. These reactions demand severe procedures that result in several disadvantages including inert reaction conditions, costly transition-metal catalysts, and time-consuming purification procedures due to the inherent formation of side products. Moreover, residues of metal catalysts remain in the HTL, which act as traps, deteriorating charge-transporting properties and negatively affecting the performance of the resulting devices.^34,35^ Therefore, the hunt is now on for new organic semiconductors that are prepared by simple, cost-effective, and green chemistry without sacrificing the efficiency.^36,37^ In this sense, enamine condensation is promising to eliminate the use of palladium-catalyzed reactions since the only byproduct is water and cost-ineffective catalysts are not involved. In addition, greatly simplified product workup and purification significantly minimize the cost of the final product.^38−40^

Herein, we present a condensation-based spirobifluorene enamine family. The influence of different substitutions in the central spirobifluorene core going from single- to multiarmed enamines was revealed, showing that a higher degree of substitution has several advantages. First, the perpendicular arrangement of the two overcrowded enamine-based molecular halves leads to a high steric demand of the resulting rigid structure, efficiently suppressing molecular interactions. Furthermore, compound **V1307** with a larger globular structure, higher molecular weight, and small intermolecular cohesion results in a high stability of the amorphous state. Comparing directly the dimethoxydiphenylamine- and bis(dimethoxydiphenylenamine)-donating fragments of spiro-OMeTAD and **V1307**, respectively, it is found that the latter has some merits. First, bis(dimethoxydiphenylenamine) being a less strong donor results in the stabilized HOMO values. Second, the more pronounced and higher degree of conjugation in enamine arms results in the better charge transport through **V1307**, a more densely packed layer. These advantages made novel enamine-derived spirobifluorene HTMs promising candidates for their successful application in PSCs, reaching a photovoltaic performance of up to 19.2% with comparable stability. With this, we demonstrate the simple enamine condensation chemistry as a universal approach to obtain highly efficient and stable HTMs.

## Results and Discussion

**Figure 1:**
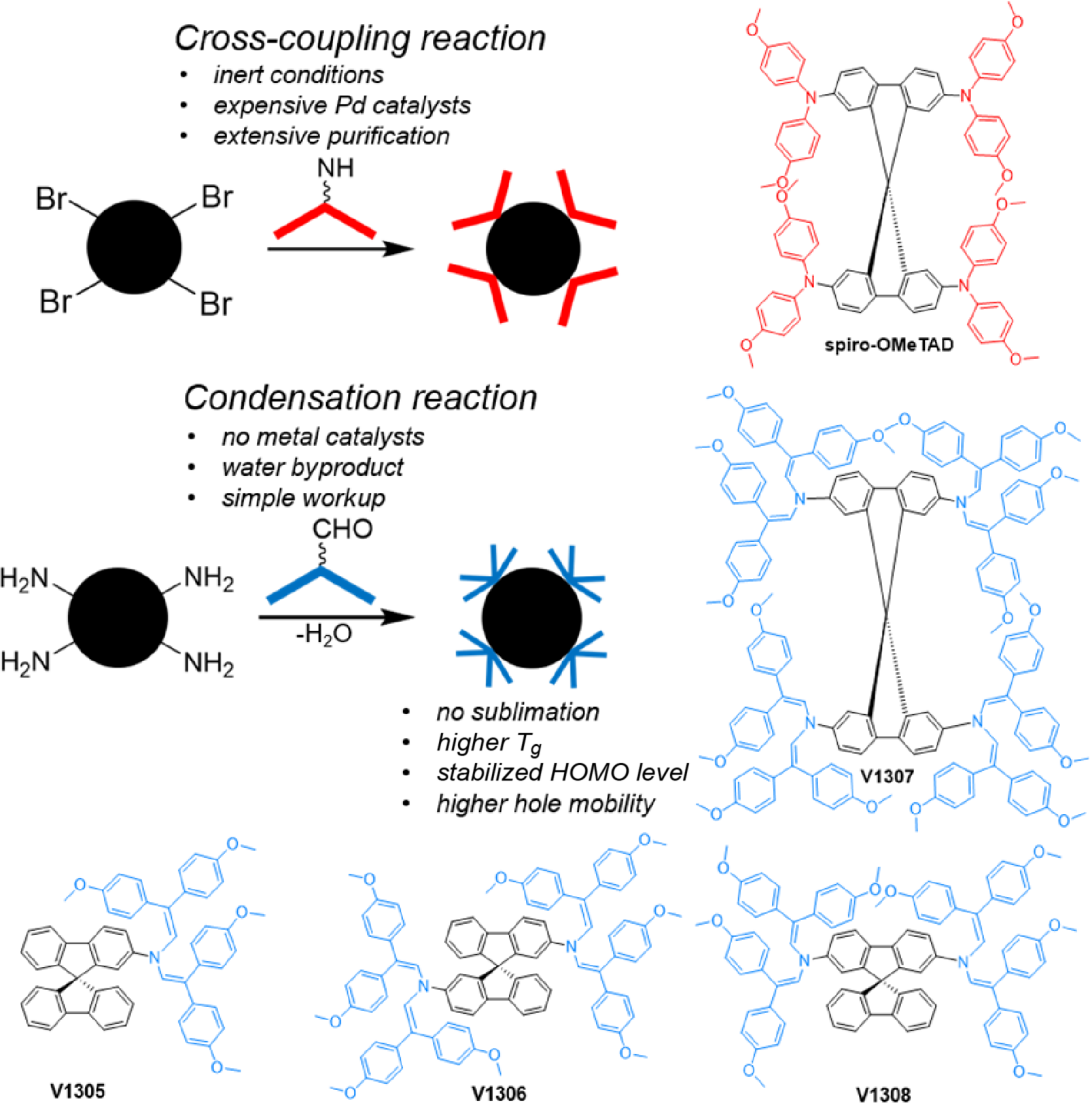
Schematic comparing cross-coupling and condensation reactions for the synthesis of spiro-OMeTAD and novel enamine HTMs.

Figure 1 presents the chemical structures of the parent spiro-OMeTAD and enamine-based analogues (**V1307**, **V1305**, **V1306**, and **V1308**). A series of four new HTMs containing a well-known spirobifluorene core with enamine arms were obtained simply by condensing aminated precursors with 2,2-bis(4-methoxyphenyl)acetaldehyde. The condensation reaction was carried out under ambient conditions, and the only byproduct is water, which is continuously separated using a Dean–Stark trap that accelerates the formation of the final product. Detailed synthetic protocols and full characterization of the compounds (NMR spectroscopy, mass spectrometry, and elemental analysis) are described in the Supporting Information. To assess the synthesis costs of the enamine condensation reaction in comparison with the regular cross-coupling reaction, we calculated the costs on a lab-scale synthesis (Table S1).^41^ The evaluated cost of **V1307** is ∼37 $/g, which is much less than the cost of spiro-OMeTAD (∼92 $/g).^42^

Thermogravimetric analysis suggests that novel HTMs start to decompose at around 400 °C (Figure S1), which is far above the temperature for conventional device operation. Differential scanning calorimetry measurements (Figure S2) indicate that **V1305**, **V1306**, and **V1308** could exist in both crystalline and amorphous states similar to spiro-OMeTAD.^43^ Interestingly, **V1307** is fully amorphous and has the highest glass-transition temperature (*T*_g_) of 169 °C, which should result in the improved quality of the **V1307** layers. We also note that all synthesized HTMs have higher *T*_g_ than spiro-OMeTAD (124 °C), meaning that the introduction of enamine fragments improves the morphological stability.

Gaussian09 software^44^ was used for simulation purposes to establish the most probable molecular geometries of **V1305**, **V1306**, **V1307**, and **V1308** with their corresponding absorption spectra. Ground state geometry optimization was performed by means of the density functional theory method with B3LYP and 6-31G basis sets without polarization functions, and the predicted molecular structures are presented in Figure S3. As expected, two fluorene core fragments (F1>C<F2) are connected through the carbon atom having a perpendicular orientation. Due to the presence of the double bonds in the enamine chain [>C=CH—N—CH=C<], orientation of substituents is chaotic and a large number of different possible positions are allowed. However, the most probable orientation is related to the V-shaped structure, when one methoxyphenyl fragment of F1 is oriented to the end of F2 (see Figure S3, **V1305**). Similar enamine branch orientations could be found in **V1306**, **V1307**, and **V1308**. Interestingly, comparing both four-armed **V1307** and spiro-OMeTAD, several differences in the molecular orientation are observed, i.e., well-ordered and dual-axed perpendicular symmetry of spiro-OMeTAD is damaged since the fluorenes in **V1307** are not completely perpendicular, and they are not flat anymore due to the chaotic arrangement of enamine substituents resulting in the fully amorphous morphology of **V1307**.

**Figure 2:**
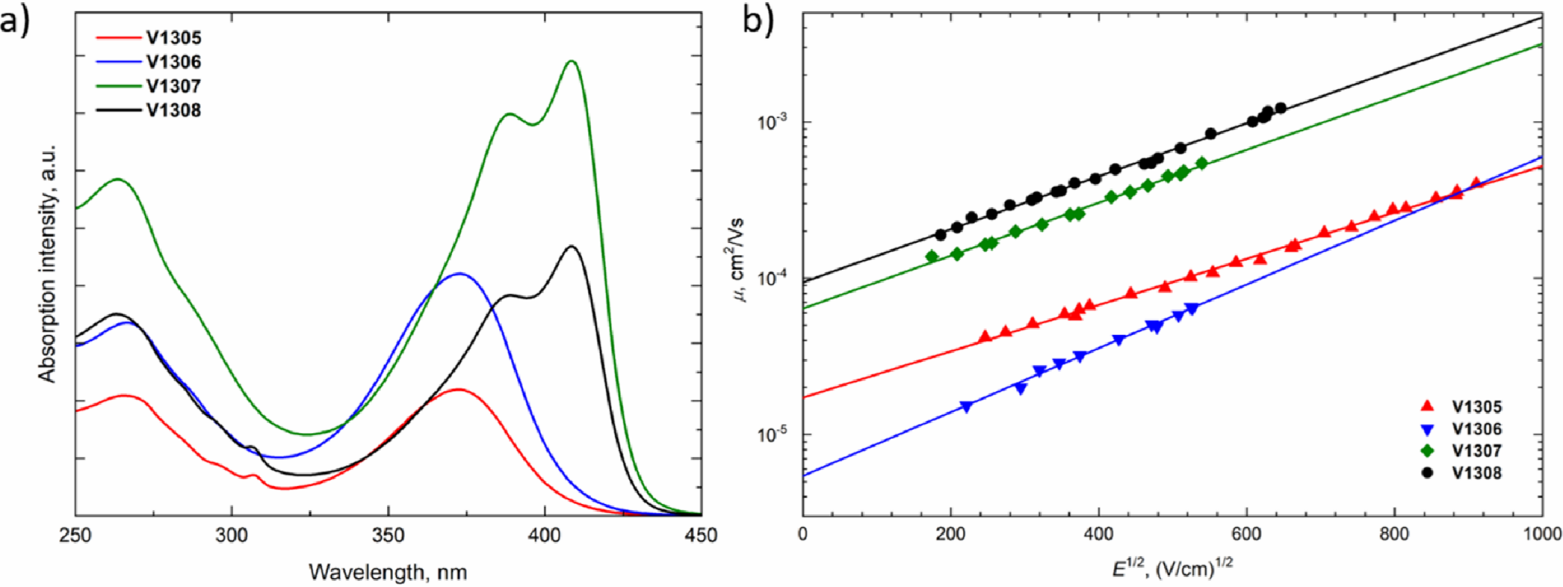
(a) UV–vis absorption spectra of **V1305**, **V1306**, **V1307**, and **V1308** in THF solutions (10^–4^ M). (b) Electric field dependencies of the hole drift mobility in spiro-enamines.

The ultraviolet–visible absorption (UV–vis) spectra in THF solutions of **V1305**, **V1306**, **V1307**, and **V1308** are shown in Figure 2a. All spiro-enamines possess two significant absorption bands at around 260 and 380 nm. The less intense absorption at shorter wavelengths corresponds to localized π–π* transitions. Longer wavelengths are observed for more intensive delocalization from the conjugated scaffold and are assigned to n−π* transitions. The change in the number and the position of enamine arms in the system has significantly influenced the absorption. While the increasing number of enamine arms shifts the absorption hyperchromically (**V1305** < **V1306** = **V1308** < **V1307**), the increased conjugation in **V1307** and **V1308**, where fluorene is substituted on both sides, also resulted in the bathochromic shift around 35 nm. The optical gaps (*E*_g_) were evaluated from the intersection of absorption and photoluminescence (PL) spectra of thin films and were estimated to be similar for all of the compounds at around 2.85 eV (Figure S6).

The solid-state ionization potentials (*I*_P_) of **V1305**, **V1306**, **V1307**, **V1308**, and spiro-OMeTAD were determined using photoemission spectroscopy in air (PESA) of the thin films to assess the HOMO energy level of spiro-enamine HTMs (Figure S7). *I*_P_ values of **V1305**, **V1306**, **V1307**, and **V1308** were found to be 5.33, 5.37, 5.46, and 5.46 eV, respectively, which are significantly stabilized compared to those of spiro-OMeTAD (5.00 eV).^40^ Based on the solid-state optical gap and *I*_P_ values, we calculated the electron affinities (*E*_ea_) to be 2.43, 2.53, 2.63, and 2.68 eV for **V1305**, **V1306**, **V1307**, and **V1308**, respectively. Importantly, the electron affinities are smaller compared with the conduction band energy of the perovskite (−4.10 eV); therefore, effective electron blocking from the perovskite to the electrode should be ensured.

Xerographic time-of-flight measurements were used to determine the charge mobility of the V-series layers. Dependencies of hole drift mobility on the electric field strength are shown in Figure 2b. **V1308** and **V1307** exhibited the highest zero-field hole-drift mobility (μ_0_) among the series with the values of 9.4 × 10^–4^ and 6.4 × 10^–4^ cm^2^/Vs, respectively, both outperforming spiro-OMeTAD (μ_0_ = 1.3 × 10^–4^ cm^2^/Vs).^34^ In addition, the lateral thin film conductivity of the V-series layers was measured with organic field-effect transistors (OFETs) (Figure S8). Similar to the result of the hole mobility measurement, **V1307** and **V1308** showed a higher conductivity (1.4 × 10^–3^ and 2.9 × 10^–4^ S cm^–1^, respectively) than **V1305** (3.9 × 10^–6^ S cm^–1^) and **V1306** (6.7 × 10^–5^ S cm^–1^). The conductivity of the doped spiro-OMeTAD as a reference was determined to be 1.0 × 10^–3^ S cm^–1^. The conductivity trend obtained for the V-series was found to be directly correlated with the degree of conjugation of the molecules. The fact that **V1307** has the higher hole mobility and conductivity value between the V-series and spiro-OMeTAD can be attributed to the higher degree of conjugation in enamine arms of **V1307**.^45−47^ The thermal, optical, and photoelectrical properties of the spiro-enamines are summarized in Table 1.

**Table 1:** Thermal, Optical, and Photophysical Properties of Newly Synthesized Enamines

ID	*T*_m_ [°C]^a^	*T*_c_ [°C]^a^	*T*_g_ [°C]^a^	*T*_dec_ [°C]^a^	λ_abs_ [nm]^b^	*I*_P_ [eV]^c^	*E*_g_ [eV]^d^	*E*_ea_ [eV]^e^	μ_0_ [cm^2^ V^–1^ s^–1^]^f^	σ [S cm^–1^]^g^
**V1305**	243		131	380	267 and 372	5.33	2.90	2.43	1.7 × 10^–5^	3.9 × 10^–6^
**V1306**	294	226	154	402	267 and 373	5.37	2.84	2.53	5.4 × 10^–6^	6.7 × 10^–5^
**V1307**			169	401	263, 389, and 408	5.46	2.83	2.63	6.4 × 10^–4^	1.4 × 10^–3^
**V1308**	305	203	158	371	263, 388, and 408	5.46	2.78	2.68	9.4 × 10^–4^	2.9 × 10^–4^

a Melting (*T*_m_), crystallization (*T*_c_), glass transition (*T*_g_), and decomposition (*T*_dec_) temperatures observed from DSC and TGA (10 °C/min, N_2_ atmosphere).

b Absorption spectra were measured in THF solutions (10^–4^ M).

c Ionization energies of the films measured using PESA.

d *E*_g_ estimated from the intersection of absorption and emission spectra of solid films.

e *E*_ea_ = *I*_P_ – *E*_g_.

f Mobility value at zero field strength.

g Conductivity values.

**Figure 3:**
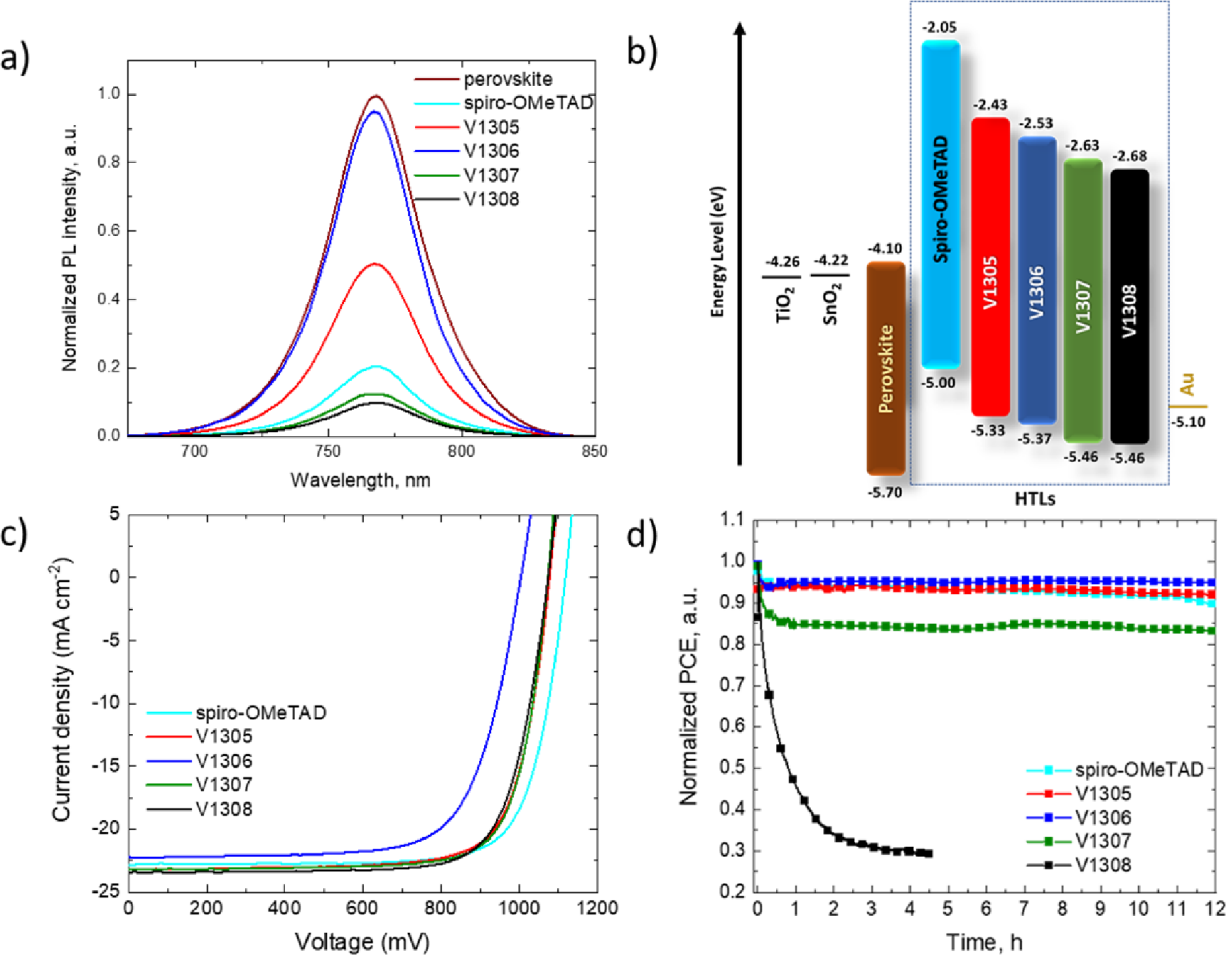
(a) PL spectra upon the 625 nm excitation wavelength of perovskite and perovskite/HTM samples deposited on glass with HTMs: spiro-OMeTAD, **V1305**, **V1306**, **V1307**, and **V1308**. PSCs with the FTO/c-TiO_2_/m-TiO_2_/SnO_2_/perovskite/HTM/Au layout: (b) energy diagram of the device, (c) *J*–*V* characteristics of the most efficient devices under 1 sun illumination measured in air, and (d) stability of unencapsulated devices under constant 1 sun illumination in the N_2_ atmosphere.

Steady-state PL was evaluated in samples with glass/perovskite and glass/perovskite/HTM layouts to determine their hole extraction properties in PSCs (Figure 3a). The PL spectra show that the hole transfer between **V1307** and **V1308** HTMs and perovskite is more efficient than that between **V1305** and **V1306**. A slight quenching effect is observed in **V1305** and **V1306**, which is in good agreement with the lowest hole drift mobility values (5.4 × 10^–6^ cm^2^/Vs for **V1305** and 1.7 × 10^–5^ cm^2^/Vs for **V1306**). This quenching is enhanced with the use of spiro-OMeTAD, **V1307**, and **V1308** HTMs that have higher hole mobility values (1.3 × 10^–4^, 6.4 × 10^–4^, and 9.4 × 10^–4^ cm^2^/Vs) due to more efficient hole transfer between perovskite and the corresponding HTM.

Fabrication of n–i–p solar cells was carried out with the device layout FTO/c-TiO_2_/m-TiO_2_/SnO_2_/perovskite/HTM/Au by spin-coating, except for the top electrode, deposited by thermal evaporation. The perovskite composition used was the triple-cation perovskite [(FAPbI_3_)_0.87_(MAPbBr_3_)_0.13_]_0.92_(CsPbI_3_)_0.08_, and the HTM is doped with the bis(trifluoromethylsulfonyl)imide lithium salt (LiTFSI) and cobalt(III)-tris(bis(trifluoromethylsulfonyl)imide). *tert*-Butylpyridine (tBP) was also added to the HTM solution to improve the HTM morphology and increase the LiTFSI solubility.^48^ The energy diagram of the device is shown in Figure 3b, and the detailed preparation process is described in the Methods section. Cross-sectional and top-view scanning electron microscopy (SEM) images were obtained to determine the thickness and morphology of the HTM, respectively (Figures S9 and S10). The HTM thickness is 180 nm for spiro-OMeTAD-based devices; 80 nm for devices containing **V1305**, **V1306**, and **V1307**; and 140 nm for devices fabricated with **V1308**.

The current–voltage (*J*–*V*) characteristics of the most efficient devices are presented in Figure 3c, and their corresponding PV parameters are extracted and shown in Table 2. The most efficient devices containing **V1305**, **V1307**, and **V1308** have similar PCE values of 19.0%, 19.2%, and 19.1%, respectively. The corresponding external quantum efficiency (EQE) and the integrated current density are shown in Figure S11. The analysis of the statistical data presented in Figure S12 and Table S4 confirms the correlation between hole-drift mobility and device performance, and being the most efficient, the devices containing the HTM with higher hole mobility values follow the order: **V1306** < **V1305** < **V1308** ∼ **V1307**.^49^

**Table 2:** PV Parameters Extracted from the Corresponding Hysteresis *J*–*V* Curves for the Best Performing Solar Cells^a^

ID		*V*_OC_ (mV)	*J*_SC_ (mA cm^–2^)	FF	PCE (%)
spiro-OMeTAD	REV	1118	22.90	0.77	19.7
	FWD	1104	22.89	0.75	19.0
**V1305**	REV	1077	23.17	0.76	19.0
	FWD	1043	23.15	0.71	17.1
**V1306**	REV	1004	22.22	0.71	15.8
	FWD	981	22.17	0.54	11.7
**V1307**	REV	1073	23.21	0.77	19.2
	FWD	1052	23.18	0.75	18.3
**V1308**	REV	1074	23.44	0.76	19.1
	FWD	1066	23.37	0.74	18.4

a REV, reverse bias; FWD, forward bias. Precondition: 2 s of light soaking. Illumination area through a shadow mask of 16 mm^2^, a scan rate of 50 mV s^–1^, and a voltage step of 10 mV.

Although the use of HTMs with deeper HOMO levels is, in principle, beneficial for the device performance due to larger *V*_OC_ values,^33,50^ in our study, all the devices showed lower *V*_OC_ values. These values (∼1070 mV) are lower compared to those of the devices containing spiro-OMeTAD (∼1100 mV) in spite of their deeper HOMO values (5.33 eV for **V1305**, 5.37 eV for **V1306**, and 5.46 for **V1307** and **V1308**) compared to spiro-OMeTAD (5.00 eV).^40^ Therefore, the lower *V*_OC_ values might be due to a contact issue at the perovskite/HTM interface producing higher charge recombination in comparison to spiro-OMeTAD-based devices. The fill factor (FF) is similar for devices containing spiro-OMeTAD, **V1305**, **V1307**, and **V1308**, but this main parameter differs for **V1306**-based devices, indicating poor film quality, which will negatively affect the hole charge extraction. The analysis of the film by top-view high-magnification SEM demonstrates the presence of pinholes in devices fabricated with **V1306** (Figure S13a). The appearance of these pinholes is also observed in the device fabricated with **V1308** (Figure S13b). However, for **V1308**, the efficiency is not affected, but it does affect the long-term device stability. The hysteretic behavior of the devices was estimated from the *J*–*V* curves collected by scanning the device from the forward bias (FB) to the short circuit (SC) followed by from SC to FB (Figure S14), and their PV parameters are reported in Table 2. The hysteresis index was calculated from the formulae previously reported,^51^ and values of 0.036, 0.100, 0.259, 0.047, and 0.037 were estimated for the devices containing spiro-OMeTAD, **V1305**, **V1306**, **V1307**, and **V1308**, respectively.

The stability of the unencapsulated devices was measured under 1 sun illumination and under a N_2_ atmosphere, as shown in Figure 3d. Devices containing **V1305** and **V1306** that have one and two enamine arms, respectively, are the most stable of the series and have a slightly better stability than devices that are composed of spiro-OMeTAD. However, the introduction of a second enamine moiety in the transconfigurated **V1308** induces a fastest decay and is completely detrimental for the stability, which is in good agreement with the inhomogeneity observed in the top-view SEM images, where the highest pinhole density was observed. Interestingly, a slight initial decay in PCE of the tetra-substituted **V1307** device was observed in the beginning of the measurement during the first hour followed by the similar trend to **V1305**, **V1306**, and spiro-OMeTAD during the remaining time. Similar dynamic mechanisms have been observed, demonstrating that a quick degradation is activated by the migration of metal electrode particles through HTM, resulting in the contact with the perovskite layer.^52,53^ Stability measurements revealed that the number and position of enamine arms in the final HTM structure have a large impact on the stability of the final device.

## Conclusions

In conclusion, a new series of spirobifluorene-based hole-transporting enamines were synthesized and systematically studied. Novel HTMs are obtained by a simple and straightforward condensation reaction, which in contrast to spiro-OMeTAD, do not require expensive palladium catalysts and inert conditions, and water is the only byproduct obtained. In addition, simple product workup and purification result in significantly reduced synthesis costs. The impact of the different number and substitution of enamine fragments was revealed through the optical, electrochemical, photophysical, and photovoltaic measurements. It was found that enamine fragments increase the glass-transition temperature of final HTMs that are more amorphous and morphological stable. Moreover, synthesized enamine materials demonstrate a very high hole mobility and conductivity up to 9.4 × 10^–4^ cm^2^/Vs and 1.4 × 10^–3^ S cm^–1^, respectively. In summary, PSCs fabricated with the novel **V1305** and **V1307** HTMs present high-efficiency values exceeding 19%, with small hysteresis and stability being comparable to those of devices containing spiro-OMeTAD in the same testing period. This demonstrates that simple enamine condensation chemistry is a universal approach to obtain highly efficient and stable HTMs.

## Methods

### Device Fabrication

Fluorine-doped tin oxide (FTO)-coated glass was laser scribed to avoid the direct contact between electrodes. The etched substrates were cleaned by the following procedure: 2% helmanex solution, deionized water, and ethanol were placed in an ultrasonic bath for 10 min and UV/O_3_ treatment was performed for 15 min. Compact TiO_2_ (c-TiO_2_) (30 nm) was deposited by spray pyrolysis from titanium diisopropoxide bis(acetylacetonate) (Sigma-Aldrich) in isopropanol solution (1:15, v/v) and annealed at 450 °C for 30 min. The mesoporous TiO_2_ (m-TiO_2_) layers were prepared from a TiO_2_ paste (Dyesol 30 NR-D) solution (1 g in 9 mL of ethanol dilution) by spin-coating at 5000 rpm (1000 rpm/s acceleration, 15 s). The layer was sintered on a hot plate at 500 °C for 30 min. Following this, a 20 nm SnO_2_ layer was prepared by dissolving SnCl_4_ (Acros) in deionized water (12 μL in 988 μL of water) and spin-coated at 3000 rpm for 30 s (1000 rpm/s acceleration), followed by annealing at 190 °C for 1 h. The [(FAPbI_3_)_0.87_(MAPbBr_3_)_0.13_]_0.92_(CsPbI_3_)_0.08_ perovskite solution was prepared by mixing 17.41 mg of MABr, 27.02 mg of CsI, 57.06 mg of PbBr_2_, 178.94 mg of FAI, and 548.60 mg of PbI_2_, and it was dissolved in 1 mL of DMF/DMSO (0.78:0.22, v/v). Then, the perovskite solution was spin-coated at 2000 rpm for 10 s, followed by 5000 rpm for 30 s. During spinning in the second step, the antisolvent chlorobenzene (110 μL) was dropped on the sample 15 s before finishing the process. The films were annealed at 100 °C for 1 h inside the glovebox. Once the samples were cooled down, the HTM was deposited on top of the perovskite layer by spin-coating at 4000 rpm for 30 s. The spiro-OMeTAD was deposited from a 60 mM solution in chlorobenzene with tBP, tris(2-(1*H*-pyrazol-1-yl)-4-*tert*-butylpyridine)cobalt(III) (FK209), and LiTFSI as additives. The concentration of V-series HTMs was 20 mM. The molar ratio of additives for spiro-OMeTAD and each doped HTM solution was 0.5 for LiTFSI from a 1.8 M stock solution in acetonitrile and 3.3 for tBP and 0.03 for FK209 from a 0.25 M stock solution in acetonitrile. Finally, a 70 nm Au film was thermally evaporated as the top electrode. The cross-sectional film morphology was investigated by using a high-resolution SEM (Merlin, Zeiss) equipped with a GEMINI II column and a Schottky Field Emission gun. Images were acquired with an In-Lens Secondary Electron Detector at 3 kV.

### Thin Film and Device Characterization

Conductivity measurements of **V1305**, **V1306**, **V1307**, **V1308**, and spiro-OMeTAD were carried out using the OFET configuration with a two-contact electrical conductivity setup. The OFET substrates were purchased from Fraunhofer IPMS. The substrates were first prepared with 20 min of oxygen plasma cleaning and subsequent film deposition of HTMs and spiro-OMeTAD by spin-coating following the same procedure as in the device fabrication, at 4000 rpm for 30 s from a chlorobenzene solution (20 mM) chemically doped with FK-209, LiTFSI, and tBP as additives. The conductivity measurements were carried out on the 2.5 μm channel by sweeping from −10 to 10 V (source–drain voltage) at a scan rate of 1 V s^–1^ with a Keithley 2612A (Figure S8). The data were recorded using the KickStart software program. The channel width and height are 10 mm and 40 nm, respectively, and the gate capacity is 15 nF. The conductivity was calculated from a linear fit of the current–voltage measurement and Ohm’s law. The *J*–*V* characteristics were determined by using a 2400 Keithley system (scan rate: 50 mV s^–1^ and 10 mV voltage step) in combination with a Xe-lamp Oriel sol3A sun simulator (Newport Corporation), previously calibrated to AM1.5G standard conditions by using the reference cell Oriel 91150 V. The devices were measured with 2 s of light soaking, and the illumination area was defined through a shadow mask of 16 mm^2^. EQE was measured with an IQE200B quantum efficiency measurement system (Oriel, Newport, UK). The stability test was performed as maximum power tracking under 100 mW cm^–2^ illumination with an LED power source. Note that during the stability test, the samples were placed in a measurement box purged continuously with nitrogen gas at 0% humidity to create an inert atmosphere. The box temperature was kept at 25 °C by a cooling system. A shadow mask of 16 mm^2^ was used to define the illumination area during the long-term stability test. Top-view SEM images were recorded by an in-lens detector of the FEI Teneo Schottky field-emission SEM at a tension of 5 kV. The steady-state PL spectra of the glass/perovskite and glass/perovskite/HTM thin films were measured and recorded using a Fluorolog-3-22 spectrofluorometer. The spectra were recorded upon excitation at 625 nm with the sample illuminated from the front side (perovskite or the HTM side).

## Supporting Information Available

Tables S1–S4 and Figures S1–S25; experimental section; detailed synthetic procedures; TGA data; DSC; optimized geometry of frontier molecular orbital diagrams; electronic absorption spectra; electronic excitation; **V1305, V1306, V1307**, and **V1308** distributions; UV–vis/PL spectra of thin films; photoemission in air spectra; current–voltage measurements; ^1^H and ^13^C NMRs; MALDI-TOF-MS spectra; PESA; conductivity; SEM images; photovoltaic parameter statistics; *J*–*V* curves; EQE (PDF)

## References

[ref1] Oga H.; Saeki A.; Ogomi Y.; Hayase S.; Seki S. Improved Understanding of the Electronic and Energetic Landscapes of Perovskite Solar Cells: High Local Charge Carrier Mobility, Reduced Recombination, and Extremely Shallow Traps. J. Am. Chem. Soc. 2014, 136, 13818–13825. 10.1021/ja506936f. 25188538

[ref2] Stranks S. D.; Eperon G. E.; Grancini G.; Menelaou C.; Alcocer M. J. P.; Leijtens T.; Herz L. M.; Petrozza A.; Snaith H. J. Electron-Hole Diffusion Lengths Exceeding 1 Micrometer in an Organometal Trihalide Perovskite Absorber. Science 2013, 342, 341–344. 10.1126/science.1243982. 24136964

[ref3] Burschka J.; Pellet N.; Moon S. J.; Humphry-Baker R.; Gao P.; Nazeeruddin M. K.; Grätzel M. Sequential Deposition as a Route to High-Performance Perovskite-Sensitized Solar Cells. Nature 2013, 499, 316–319. 10.1038/nature12340. 23842493

[ref4] Jeon N. J.; Noh J. H.; Yang W. S.; Kim Y. C.; Ryu S.; Seo J.; Seok S. I. Compositional Engineering of Perovskite Materials for High-Performance Solar Cells. Nature 2015, 517, 476–480. 10.1038/nature14133. 25561177

[ref5] Saliba M.; Matsui T.; Seo J. Y.; Domanski K.; Correa-Baena J. P.; Nazeeruddin M. K.; Zakeeruddin S. M.; Tress W.; Abate A.; Hagfeldt A.; Grätzel M. Cesium-Containing Triple Cation Perovskite Solar Cells: Improved Stability, Reproducibility and High Efficiency. Energy Environ. Sci. 2016, 9, 1989–1997. 10.1039/C5EE03874J. 27478500 PMC4936376

[ref6] Wehrenfennig C.; Eperon G. E.; Johnston M. B.; Snaith H. J.; Herz L. M. High Charge Carrier Mobilities and Lifetimes in Organolead Trihalide Perovskites. Adv. Mater. 2014, 26, 1584–1589. 10.1002/adma.201305172. 24757716 PMC4722848

[ref7] Lee M. M.; Teuscher J.; Miyasaka T.; Murakami T. N.; Snaith H. J. Efficient Hybrid Solar Cells Based on Meso-Superstructured Organometal Halide Perovskites. Science 2012, 338, 643–647. 10.1126/science.1228604. 23042296

[ref8] Zhao Y.; Zhu K. Organic-Inorganic Hybrid Lead Halide Perovskites for Optoelectronic and Electronic Applications. Chem. Soc. Rev. 2016, 45, 655–689. 10.1039/C4CS00458B. 26645733

[ref9] Liu M.; Johnston M. B.; Snaith H. J. Efficient Planar Heterojunction Perovskite Solar Cells by Vapour Deposition. Nature 2013, 501, 395–398. 10.1038/nature12509. 24025775

[ref10] Jeon N. J.; Noh J. H.; Kim Y. C.; Yang W. S.; Ryu S.; Seok S. I. Solvent Engineering for High-Performance Inorganic-Organic Hybrid Perovskite Solar Cells. Nat. Mater. 2014, 13, 897–903. 10.1038/nmat4014. 24997740

[ref11] Bach U.; Lupo D.; Comte P.; Moser J. E.; Weissörtel F.; Salbeck J.; Spreitzer H.; Grätzel M. Solid-State Dye-Sensitized Mesoporous TiO2 Solar Cells with High Photon-to-Electron Conversion Efficiencies. Nature 1998, 395, 583–585. 10.1038/26936.

[ref12] Jiang K.; Wu F.; Zhang G.; Zhu L.; Yan H. Efficient Perovskite Solar Cells Based on Dopant-Free Spiro-OMeTAD Processed With Halogen-Free Green Solvent. Sol. RRL 2019, 3, 1900061 10.1002/solr.201900061.

[ref13] Luo W.; Wu C.; Wang D.; Zhang Z.; Qi X.; Guo X.; Qu B.; Xiao L.; Chen Z. Dopant-Free Spiro-OMeTAD as Hole Transporting Layer for Stable and Efficient Perovskite Solar Cells. Org. Electron. 2019, 74, 7–12. 10.1016/j.orgel.2019.06.039.

[ref14] Rakstys K.; Igci C.; Nazeeruddin M. K. Efficiency: Vs. Stability: Dopant-Free Hole Transporting Materials towards Stabilized Perovskite Solar Cells. Chem. Sci. 2019, 10, 6748–6769. 31391896 10.1039/c9sc01184f PMC6657418

[ref15] Hawash Z.; Ono L. K.; Qi Y. Recent Advances in Spiro-MeOTAD Hole Transport Material and Its Applications in Organic–Inorganic Halide Perovskite Solar Cells. Adv. Mater. Interfaces 2018, 5, 1700623 10.1002/admi.201700623.

[ref16] Gangala S.; Misra R. Spiro-Linked Organic Small Molecules as Hole-Transport Materials for Perovskite Solar Cells. J. Mater. Chem. A 2018, 6, 18750–18765. 10.1039/C8TA08503J.

[ref17] Saragi T. P. I.; Spehr T.; Siebert A.; Fuhrmann-Lieker T.; Salbeck J. Spiro Compounds for Organic Optoelectronics. Chem. Rev. 2007, 107, 1011–1065. 10.1021/cr0501341. 17381160

[ref18] Jeon N. J.; Na H.; Jung E. H.; Yang T. Y.; Lee Y. G.; Kim G.; Shin H. W.; Il Seok S.; Lee J.; Seo J. A Fluorene-Terminated Hole-Transporting Material for Highly Efficient and Stable Perovskite Solar Cells. Nat. Energy 2018, 3, 682–689. 10.1038/s41560-018-0200-6.

[ref19] Chen C. C.; Deng Z.; He M.; Zhang Y.; Ullah F.; Ding K.; Liang J.; Zhang Z.; Xu H.; Qiu Y.; Xie Z.; Shan T.; Chen Z.; Zhong H. Design of Low Crystallinity Spiro-Typed Hole Transporting Material for Planar Perovskite Solar Cells to Achieve 21.76% Efficiency. Chem. Mater. 2021, 33, 285–297. 10.1021/acs.chemmater.0c03772.

[ref20] Jeong M.; Choi I. W.; Go E. M.; Cho Y.; Kim M.; Lee B.; Jeong S.; Jo Y.; Choi H. W.; Lee J.; Bae J. H.; Kwak S. K.; Kim D. S.; Yang C. Stable Perovskite Solar Cells with Efficiency Exceeding 24.8% and 0.3-V Voltage Loss. Science 2020, 369, 1615–1620. 10.1126/science.abb7167. 32973026

[ref21] Bi D.; Xu B.; Gao P.; Sun L.; Grätzel M.; Hagfeldt A. Facile Synthesized Organic Hole Transporting Material for Perovskite Solar Cell with Efficiency of 19.8%. Nano Energy 2016, 23, 138–144. 10.1016/j.nanoen.2016.03.020.

[ref22] Xu B.; Bi D.; Hua Y.; Liu P.; Cheng M.; Grätzel M.; Kloo L.; Hagfeldt A.; Sun L. A Low-Cost Spiro[Fluorene-9,9′-Xanthene]-Based Hole Transport Material for Highly Efficient Solid-State Dye-Sensitized Solar Cells and Perovskite Solar Cells. Energy Environ. Sci. 2016, 9, 873–877. 10.1039/C6EE00056H.

[ref23] Zhang J.; Xu B.; Yang L.; Ruan C.; Wang L.; Liu P.; Zhang W.; Vlachopoulos N.; Kloo L.; Boschloo G.; Sun L.; Hagfeldt A.; Johansson E. M. J. The Importance of Pendant Groups on Triphenylamine-Based Hole Transport Materials for Obtaining Perovskite Solar Cells with over 20% Efficiency. Adv. Energy Mater. 2018, 8, 1701209 10.1002/aenm.201701209.

[ref24] Xu B.; Zhang J.; Hua Y.; Liu P.; Wang L.; Ruan C.; Li Y.; Boschloo G.; Johansson E. M. J.; Kloo L.; Hagfeldt A.; Jen A. K. Y.; Sun L. Tailor-Making Low-Cost Spiro[Fluorene-9,9′-Xanthene]-Based 3D Oligomers for Perovskite Solar Cells. Chem. 2017, 2, 676–687. 10.1016/j.chempr.2017.03.011.

[ref25] Chiykowski V. A.; Cao Y.; Tan H.; Tabor D. P.; Sargent E. H.; Aspuru-Guzik A.; Berlinguette C. P. Precise Control of Thermal and Redox Properties of Organic Hole-Transport Materials. Angew. Chem., Int. Ed. 2018, 57, 15529–15533. 10.1002/anie.201810809. 30267466

[ref26] Drigo N.; Roldan-Carmona C.; Franckevičius M.; Lin K. H.; Gegevičius R.; Kim H.; Schouwink P. A.; Sutanto A. A.; Olthof S.; Sohail M.; Meerholz K.; Gulbinas V.; Corminboeuf C.; Paek S.; Nazeeruddin M. K. Doped but Stable: Spirobisacridine Hole Transporting Materials for Hysteresis-Free and Stable Perovskite Solar Cells. J. Am. Chem. Soc. 2020, 142, 1792–1800. 10.1021/jacs.9b07166. 31865703

[ref27] Saliba M.; Orlandi S.; Matsui T.; Aghazada S.; Cavazzini M.; Correa-Baena J. P.; Gao P.; Scopelliti R.; Mosconi E.; Dahmen K. H.; De Angelis F.; Abate A.; Hagfeldt A.; Pozzi G.; Graetzel M.; Nazeeruddin M. K. A Molecularly Engineered Hole-Transporting Material for Efficient Perovskite Solar Cells. Nat. Energy 2016, 1, 15017 10.1038/nenergy.2015.17.

[ref28] Rakstys K.; Paek S.; Sohail M.; Gao P.; Cho K. T.; Gratia P.; Lee Y.; Dahmen K. H.; Nazeeruddin M. K. A Highly Hindered Bithiophene-Functionalized Dispiro-Oxepine Derivative as an Efficient Hole Transporting Material for Perovskite Solar Cells. J. Mater. Chem. A 2016, 4, 18259–18264. 10.1039/C6TA09028A.

[ref29] Gao K.; Xu B.; Hong C.; Shi X.; Liu H.; Li X.; Xie L.; Jen A. K. Y. Di-Spiro-Based Hole-Transporting Materials for Highly Efficient Perovskite Solar Cells. Adv. Energy Mater. 2018, 8, 1800809 10.1002/aenm.201800809.

[ref30] Zhu X. D.; Ma X. J.; Wang Y. K.; Li Y.; Gao C. H.; Wang Z. K.; Jiang Z. Q.; Liao L. S. Hole-Transporting Materials Incorporating Carbazole into Spiro-Core for Highly Efficient Perovskite Solar Cells. Adv. Funct. Mater. 2019, 29, 1807094.

[ref31] Wang X.; Zhang J.; Yu S.; Yu W.; Fu P.; Liu X.; Tu D.; Guo X.; Li C. Lowering Molecular Symmetry To Improve the Morphological Properties of the Hole-Transport Layer for Stable Perovskite Solar Cells. Angew. Chem. 2018, 130, 12709–12713. 10.1002/ange.201807402. 30076685

[ref32] Truong M. A.; Lee J.; Nakamura T.; Seo J. Y.; Jung M.; Ozaki M.; Shimazaki A.; Shioya N.; Hasegawa T.; Murata Y.; Zakeeruddin S. M.; Grätzel M.; Murdey R.; Wakamiya A. Influence of Alkoxy Chain Length on the Properties of Two-Dimensionally Expanded Azulene-Core-Based Hole-Transporting Materials for Efficient Perovskite Solar Cells. Chem. – Eur. J. 2019, 25, 6741–6752. 10.1002/chem.201806317. 30805960

[ref33] Rakstys K.; Abate A.; Dar M. I.; Gao P.; Jankauskas V.; Jacopin G.; Kamarauskas E.; Kazim S.; Ahmad S.; Grätzel M.; Nazeeruddin M. K. Triazatruxene-Based Hole Transporting Materials for Highly Efficient Perovskite Solar Cells. J. Am. Chem. Soc. 2015, 137, 16172–16178. 10.1021/jacs.5b11076. 26630459

[ref34] Rakstys K.; Saliba M.; Gao P.; Gratia P.; Kamarauskas E.; Paek S.; Jankauskas V.; Nazeeruddin M. K. Highly Efficient Perovskite Solar Cells Employing an Easily Attainable Bifluorenylidene-Based Hole-Transporting Material. Angew. Chem., Int. Ed. 2016, 55, 7464–7468. 10.1002/anie.201602545. 27158924

[ref35] Usluer Ö.; Abbas M.; Wantz G.; Vignau L.; Hirsch L.; Grana E.; Brochon C.; Cloutet E.; Hadziioannou G. Metal Residues in Semiconducting Polymers: Impact on the Performance of Organic Electronic Devices. ACS Macro Lett. 2014, 3, 1134–1138. 10.1021/mz500590d. 35610811

[ref36] Petrus M. L.; Music A.; Closs A. C.; Bijleveld J. C.; Sirtl M. T.; Hu Y.; Dingemans T. J.; Bein T.; Docampo P. Design Rules for the Preparation of Low-Cost Hole Transporting Materials for Perovskite Solar Cells with Moisture Barrier Properties. J. Mater. Chem. A 2017, 5, 25200–25210. 10.1039/C7TA06452G.

[ref37] Petrus M. L.; Schutt K.; Sirtl M. T.; Hutter E. M.; Closs A. C.; Ball J. M.; Bijleveld J. C.; Petrozza A.; Bein T.; Dingemans T. J.; Savenije T. J.; Snaith H.; Docampo P. New Generation Hole Transporting Materials for Perovskite Solar Cells: Amide-Based Small-Molecules with Nonconjugated Backbones. Adv. Energy Mater. 2018, 8, 1–11. 10.1002/aenm.201801605.

[ref38] Daskeviciene M.; Paek S.; Wang Z.; Malinauskas T.; Jokubauskaite G.; Rakstys K.; Cho K. T.; Magomedov A.; Jankauskas V.; Ahmad S.; Snaith H. J.; Getautis V.; Nazeeruddin M. K. Carbazole-Based Enamine: Low-Cost and Efficient Hole Transporting Material for Perovskite Solar Cells. Nano Energy 2017, 32, 551–557. 10.1016/j.nanoen.2017.01.015.

[ref39] Vaitukaityte D.; Wang Z.; Malinauskas T.; Magomedov A.; Bubniene G.; Jankauskas V.; Getautis V.; Snaith H. J. Efficient and Stable Perovskite Solar Cells Using Low-Cost Aniline-Based Enamine Hole-Transporting Materials. Adv. Mater. 2018, 30, 1–7. 10.1002/adma.201803735. 30247784

[ref40] Daskeviciute S.; Momblona C.; Rakstys K.; Sutanto A. A.; Daskeviciene M.; Jankauskas V.; Gruodis A.; Bubniene G.; Getautis V.; Nazeeruddin M. K. Fluorene-Based Enamines as Low-Cost and Dopant-Free Hole Transporting Materials for High Performance and Stable Perovskite Solar Cells. J. Mater. Chem. A 2021, 9, 301–309. 10.1039/D0TA08452B.

[ref41] Osedach T. P.; Andrew T. L.; Bulović V. Effect of Synthetic Accessibility on the Commercial Viability of Organic Photovoltaics. Energy Environ. Sci. 2013, 6, 711–718. 10.1039/c3ee24138f.

[ref42] Petrus M. L.; Bein T.; Dingemans T. J.; Docampo P.; Low Cost A. Azomethine-Based Hole Transporting Material for Perovskite Photovoltaics. J. Mater. Chem. A 2015, 3, 12159–12162. 10.1039/C5TA03046C.

[ref43] Malinauskas T.; Tomkute-Luksiene D.; Sens R.; Daskeviciene M.; Send R.; Wonneberger H.; Jankauskas V.; Bruder I.; Getautis V. Enhancing Thermal Stability and Lifetime of Solid-State Dye-Sensitized Solar Cells via Molecular Engineering of the Hole-Transporting Material Spiro-OMeTAD. ACS Appl. Mater. Interfaces 2015, 7, 11107–11116. 10.1021/am5090385. 25954820

[ref44] Additional Citation Recommendations. Gaussian 09 Citation Gaussian.com, https://gaussian.com/g09citation/ (accessed 2020-05-15).

[ref45] Liao Y. L.; Hung W. Y.; Hou T. H.; Lin C. Y.; Wong K. T. Hole Mobilities of 2,7- And 2,2-Disubstituted 9,9′-Spirobifluorene-Based Triaryldiamines and Their Application as Hole Transport Materials in OLEDs. Chem. Mater. 2007, 19, 6350–6357. 10.1021/cm702230e.

[ref46] Qiu J.; Liu H.; Li X.; Wang S. Position Effect of Arylamine Branches on Pyrene-Based Dopant-Free Hole Transport Materials for Efficient and Stable Perovskite Solar Cells. Chem. Eng. J. 2020, 387, 123965 10.1016/j.cej.2019.123965.

[ref47] Liu P.; Xu B.; Hua Y.; Cheng M.; Aitola K.; Sveinbjörnsson K.; Zhang J.; Boschloo G.; Sun L.; Kloo L. Design, Synthesis and Application of a Π-Conjugated, Non-Spiro Molecular Alternative as Hole-Transport Material for Highly Efficient Dye-Sensitized Solar Cells and Perovskite Solar Cells. J. Power Sources 2017, 344, 11–14. 10.1016/j.jpowsour.2017.01.092.

[ref48] Wang S.; Sina M.; Parikh P.; Uekert T.; Shahbazian B.; Devaraj A.; Meng Y. S. Role of 4-Tert-Butylpyridine as a Hole Transport Layer Morphological Controller in Perovskite Solar Cells. Nano Lett. 2016, 16, 5594–5600. 10.1021/acs.nanolett.6b02158. 27547991

[ref49] Daskeviciene M.; Paek S.; Magomedov A.; Cho K. T.; Saliba M.; Kizeleviciute A.; Malinauskas T.; Gruodis A.; Jankauskas V.; Kamarauskas E.; Nazeeruddin M. K.; Getautis V. Molecular Engineering of Enamine-Based Small Organic Compounds as Hole-Transporting Materials for Perovskite Solar Cells. J. Mater. Chem. C 2019, 7, 2717–2724. 10.1039/C8TC06297H.

[ref50] Urbani M.; De La Torre G.; Nazeeruddin M. K.; Torres T. Phthalocyanines and Porphyrinoid Analogues as Hole-and Electron-Transporting Materials for Perovskite Solar Cells. Chem. Soc. Rev. 2019, 48, 2738–2766. 10.1039/C9CS00059C. 31033978

[ref51] Habisreutinger S. N.; Noel N. K.; Snaith H. J. Hysteresis Index: A Figure without Merit for Quantifying Hysteresis in Perovskite Solar Cells. ACS Energy Lett. 2018, 3, 2472–2476. 10.1021/acsenergylett.8b01627.

[ref52] Guarnera S.; Abate A.; Zhang W.; Foster J. M.; Richardson G.; Petrozza A.; Snaith H. J. Improving the Long-Term Stability of Perovskite Solar Cells with a Porous Al2O3 Buffer Layer. J. Phys. Chem. Lett. 2015, 6, 432–437. 10.1021/jz502703p. 26261960

[ref53] Domanski K.; Correa-Baena J. P.; Mine N.; Nazeeruddin M. K.; Abate A.; Saliba M.; Tress W.; Hagfeldt A.; Grätzel M. Not All That Glitters Is Gold: Metal-Migration-Induced Degradation in Perovskite Solar Cells. ACS Nano 2016, 10, 6306–6314. 10.1021/acsnano.6b02613. 27187798

