# Supplementary material for: Cut from the Same Cloth: Enamine-Derived Spirobifluorenes as Hole Transporters for Perovskite Solar Cells
Source: Chem Mater. 2021 Jul 19;33(15):6059–67. doi: 10.1021/acs.chemmater.1c01486 (PMC8382250; doi:10.1021/acs.chemmater.1c01486)
Supplement: Supplementary file 1 — cm1c01486_si_001.pdf [file cm1c01486_si_001.pdf]

Supplementary Information

Cut from the same cloth: enamine-derived spirobifluorenes as hole transporters for perovskite solar cells

Deimante Vaitukaityte, Cristina Momblona, Kasparas Rakstys, Albertus Adrian Sutanto, Bin Ding, Cansu Igci, Vygintas Jankauskas, Alytis Gruodis, Tadas Malinauskas, Abdullah M. Asiri, Paul J. Dyson, Vytautas Getautis,* Mohammad Khaja Nazeeruddin**

EXPERIMENTAL SECTION

Chemicals required for the synthesis were purchased from Sigma-Aldrich and TCI Europe and used as received without additional purification. 9,9'-spirobi[fluorene]-2,2'-diamine and 2,2',7,7'-tetraamino-9,9'-spirobifluorene were purchased from Fluorochem and Sagechem, respectively. 9,9'-spirobi[fluorene]-2,7-diamine was synthesized following the literature procedure.[1] ^{1}H NMR spectra were recorded at 400 MHz on a Bruker Avance III spectrometer with a 5 mm double resonance broad band BBO z-gradient room temperature probe, ^{13}C NMR spectra were collected using the same instrument at 101 MHz. The chemical shifts, expressed in ppm, were relative to tetramethylsilane (TMS). All the NMR experiments were performed at 25 °C. Reactions were monitored by thin-layer chromatography on ALUGRAM SIL G/UV254 plates and developed with UV light. Silica gel (grade 9385, 230–400 mesh, 60 Å, Aldrich) was used for column chromatography. Elemental analysis was performed with an Exeter Analytical CE-440 elemental analyser, Model 440 C/H/N/. MS were recorded on Thermo Fisher Q Exactive HF Hybrid Quadrupole-Orbitrap Mass Spectrometer using matrix-assisted laser desorption/ionization (MALDI) technique.

DETAILED SYNTHETIC PROCEDURES

2,2-bis(4-methoxyphenyl)acetaldehyde

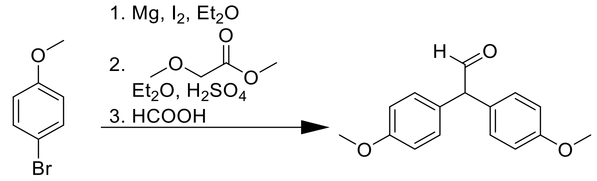

4-bromoanisole (38.2 ml, 305 mmol) was dissolved in dry diethyl ether (102 ml) under argon atmosphere. Separately, magnesium turnings (7.4 g, 305 mmol) were added to dry diethyl ether (60 ml) and activated with iodine crystal and the solution of 4-bromoanisole was added dropwise at such a rate to maintain a steady reflux. After the addition, the resulting mixture was refluxed for 1 hour under argon atmosphere and cooled down to RT. Methyl methoxyacetate (9.9 ml, 100 mmol) was dissolved in dry diethyl ether (50 ml) separately and the following solution was added to the Grignard reagent dropwise at such a rate that gentle reflux was maintained. After the addition, resulting mixture was heated to reflux for 1 hour and the reaction flask was filled with ice and 49.5 ml of sulfuric acid solution (33 %, v/v) and stirred for 5 min. The reaction mixture was extracted with diethyl ether. The organic layer was dried over anhydrous Na_2SO_4, filtered and solvent evaporated. The crude compound was refluxed in 85 ml of formic acid for 6 hours under argon atmosphere. The reaction mixture was extracted with ethyl acetate, the organic layer was dried over anhydrous Na_2SO_4, filtered and solvent evaporated. The final product was crystallized from diethyl ether/ethanol 1:1 (30 ml), filtered off and washed with ethanol two times (14.5 g, 23%). ^{1}H NMR (400 MHz, THF-d_8) δ: 9.82 (d, J = 2.6 Hz, 1H); 7.13 (d, J = 8.6 Hz, 4H); 6.88 (d, J = 8.7 Hz, 4H); 4.77 (d, J = 2.6 Hz, 1H); 3.75 (s, 6H) ppm. ^{13}C NMR (101 MHz, THF) δ: 198.43; 160.12; 130.93; 130.30; 114.95; 63.27; 55.44 ppm.

***N,N*-bis[2,2-bis(4-methoxyphenyl)vinyl]-9,9'-spirobi[fluoren]-2-amine (V1305)**

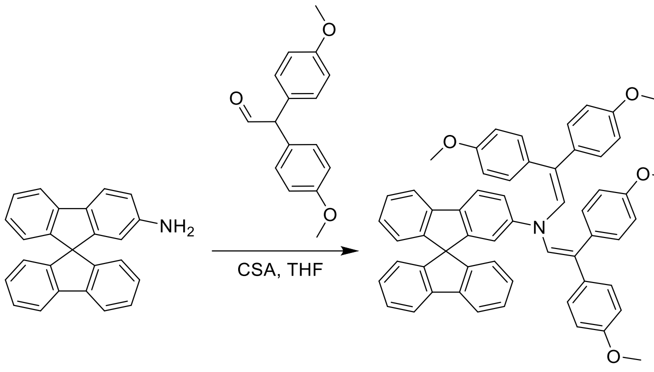

9,9'-spirobi[9H-fluoren]-2-amine (0.30 g, 0.91 mmol) was dissolved in tetrahydrofuran (9 mL + volume of the Dean-Stark trap), (+/-)camphor-10-sulphonic acid (0.21 g, 0.91 mmol) was added and the mixture was heated at reflux for 20 minutes. Afterwards, 2,2-bis(4-methoxyphenyl)acetaldehyde (0.70 g, 2.72 mmol) was added, and reflux continued using a Dean-Stark trap. After termination of the reaction (6 h, TLC, THF:*n*-hexane, 7:18) the reaction mixture was extracted with ethyl acetate. The organic layer was dried over anhydrous Na_2SO_4, filtered and solvent evaporated. The crude product was purified by column chromatography using THF:*n*-hexane (v:v; 4:21) eluent. The obtained product was precipitated from THF into 20-fold excess of methanol. The precipitate was filtered off and washed with methanol to collect **V1305** as a yellow solid (0.36 g, 49%). ^{1}H NMR (400 MHz, THF-*d$_8$*) δ: 7.83 (d, *J* = 8.3 Hz, 1H); 7.79 (d, *J* = 7.6 Hz, 3H); 7.33 – 7.21 (m, 3H); 7.16 (dd, *J* = 8.3, 2.2 Hz, 1H); 7.09 (t, *J* = 6.9 Hz, 2H); 6.94 (t, *J* = 6.9 Hz, 1H); 6.85 (d, *J* = 8.7 Hz, 4H); 6.77 (d, *J* = 8.8 Hz, 4H); 6.72 (d, *J* = 7.6 Hz, 2H); 6.53 (d, *J* = 8.8 Hz, 4H); 6.50 – 6.44 (m, 2H); 6.33 (d, *J* = 8.7 Hz, 4H); 5.60 (s, 2H); 3.78 (s, 6H); 3.66 (s, 6H) ppm. ^{13}C NMR (101 MHz, THF) δ: 160.44; 160.13; 151.16; 150.20; 150.12; 147.58; 142.88; 142.62; 137.27; 135.19; 133.62; 132.08; 131.50; 129.83; 128.66; 128.62; 128.49; 127.57; 127.49; 124.89; 124.41; 121.55; 121.01; 120.09; 118.07; 114.75; 113.76; 112.91; 66.50; 55.69; 55.44 ppm. Anal. calcd for $C_{57}H_{45}NO_4$: C 84.73; H 5.61; N 1.73; found: C 84.59; H 5.69; N 1.92. $C_{57}H_{45}NO_4$[M$^+$] exact mass = 807.335, MS (MALDI-TOF) = 807.391.

***N*[2],*N*[2],*N*[2'],*N*[2']-tetrakis[2,2-bis(4-methoxyphenyl)vinyl]-9,9'-spirobi[fluorene]-2,2'-diamine (V1306)**

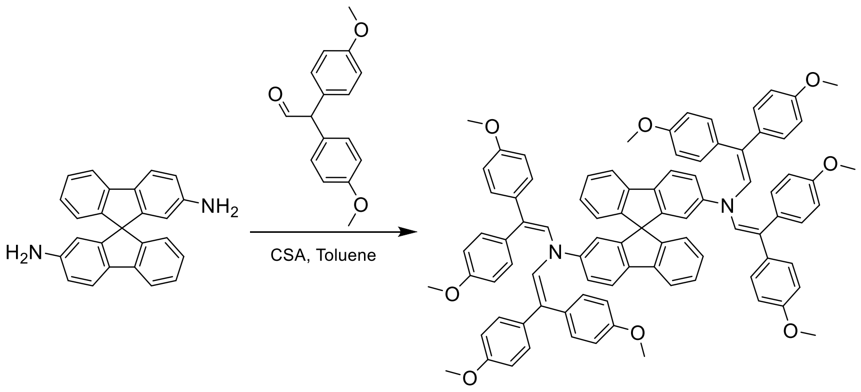

9,9'-spirobi[fluorene]-2,2'-diamine (0.30 g, 0.87 mmol) was dissolved in toluene (9 mL + volume of the Dean-Stark trap), (+/-)camphor-10-sulphonic acid (0.20 g, 0.87 mmol) was added and the mixture was heated at reflux for 20 minutes. Afterwards, 2,2-bis(4-methoxyphenyl)acetaldehyde (1.33 g, 5.2 mmol) was added, and reflux continued using a Dean-Stark trap. After termination of the reaction (6 h, TLC, THF:*n*-hexane, 2:3) the reaction mixture was poured into 15-fold excess of ethanol. The product was filtered off and washed with ethanol. The crude product was purified by column chromatography using THF:*n*-hexane (v:v; 7:18) eluent. The obtained product was precipitated from THF into 15-fold excess of methanol. The precipitate was filtered off and washed with methanol to collect **V1306** as a yellow solid (0.65 g, 58%). ^{1}H NMR (400 MHz, THF-d_8) δ: 7.74 (d, *J* = 8.3 Hz, 2H); 7.70 (d, *J* = 7.6 Hz, 2H); 7.21 (t, *J* = 7.0 Hz, 2H); 7.10 (dd, *J* = 8.3, 2.2 Hz, 2H); 6.96 (t, *J* = 7.5 Hz, 2H); 6.89 (d, *J* = 8.8 Hz, 8H); 6.79 (d, *J* = 8.8 Hz, 8H); 6.62 – 6.52 (m, 12H); 6.36 (d, *J* = 8.8 Hz, 8H); 5.63 (s, 4H); 3.80 (s, 12H); 3.69 (s, 12H) ppm. ^{13}C NMR (101 MHz; THF) δ: 160.48; 160.17; 151.32; 150.51; 147.54; 142.40; 137.19; 135.13; 133.73; 132.28; 131.50; 129.88; 128.44; 127.69; 127.46; 124.33; 121.64; 120.08; 117.85; 114.90; 113.81; 112.52; 66.50; 55.72; 55.46 ppm. Anal. calcd for $C_{89}H_{74}N_2O_8$: C 82.26; H 5.74; N 2.16; found: C 79.33; H 6.66; N 2.08. $C_{89}H_{74}N_2O_8$[M^+] exact mass = 1298.545, MS (MALDI-TOF) = 1299.539.

N^2,N^2,$N^{2'}$,$N^{2'}$,N^7,N^7,$N^{7'}$,$N^{7'}$-octakis[2,2-bis(4-methoxyphenyl)vinyl]-9,9'-spirobi[fluorene]-2,2',7,7'-tetraamine (V1307)

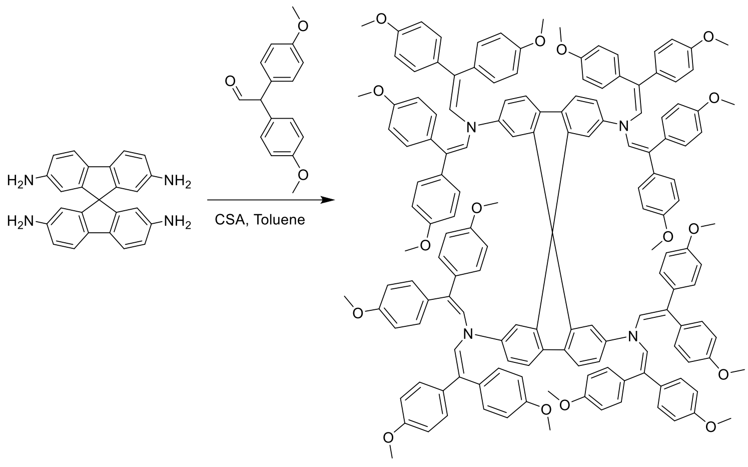

2,2',7,7'-Tetraamino-9,9'-spirobifluorene (0.20 g, 0.53 mmol) was dissolved in toluene (7 mL + volume of the Dean-Stark trap), (+/-)camphor-10-sulphonic acid (0.12 g, 0.53 mmol) was added and the mixture was heated at reflux for 20 minutes. Afterwards, 2,2-bis(4-methoxyphenyl)acetaldehyde (1.63 g, 6.36 mmol) was added, and reflux continued using a Dean-Stark trap. After termination of the reaction (5 h, TLC, acetone:*n*-hexane, 2:3) the reaction mixture was poured into 15-fold excess of methanol. The product was filtered off and washed with methanol. The crude product was purified by column chromatography using THF:*n*-hexane (v:v; 11:14) eluent. The obtained product was precipitated from THF into 15-fold excess of methanol. The precipitate was filtered off and washed with methanol to collect **V1307** as a yellow solid (0.4 g, 33%). ^{1}H NMR (400 MHz, THF-d_8) δ: 7.55 (d, J = 8.3 Hz, 4H); 6.98 (dd, J = 8.3, 2.2 Hz, 4H); 6.91 (d, J = 8.7 Hz, 16H); 6.83 (d, J = 8.8 Hz, 16H); 6.61 (d, J = 8.8 Hz, 16H); 6.50 (d, J = 2.2 Hz, 4H); 6.40 (d, J = 8.8 Hz, 16H); 5.65 (s, 8H); 3.86 (s, 24H); 3.70 (s, 24H) ppm. ^{13}C NMR (101 MHz, THF) δ: 160.49; 160.13; 151.90; 146.56; 136.85; 135.24; 133.90; 132.05; 131.53; 129.94; 127.92; 120.79; 117.79; 114.97; 113.82; 112.19; 66.50; 55.78; 55.45 ppm. Anal. calcd for $C_{153}H_{132}N_4O_{16}$: C 80.50; H 5.83; N 2.45; found: C 77.9; H 5.7; N 2.46. $C_{153}H_{132}N_4O_{16}$[M^+] exact mass = 2281.967, MS (MALDI-TOF) = 2282.119.

***N*[2],*N*[2],*N*[7],*N*[7]-tetrakis[2,2-bis(4-methoxyphenyl)vinyl]-9,9'-spirobi[fluorene]-2,7-diamine (V1308)**

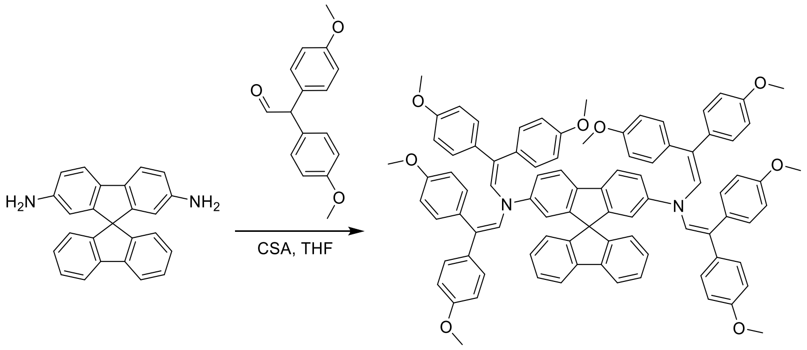

9,9'-spirobi[fluorene]-2,7-diamine[1] (0.14 g, 0.42 mmol) was dissolved in toluene (5 mL + volume of the Dean-Stark trap), (+/-)camphor-10-sulphonic acid (0.10 g, 0.42 mmol) was added and the mixture was heated at reflux for 20 minutes. Afterwards, 2,2-bis(4-methoxyphenyl)acetaldehyde (0.64 g, 2.50 mmol) was added, and reflux continued using a Dean-Stark trap. After termination of the reaction (5 h, TLC, acetone:*n*-hexane, 7:18) the reaction mixture was extracted with ethyl acetate. The organic layer was dried over anhydrous Na_2SO_4, filtered and solvent evaporated. The crude product was purified by column chromatography using THF:*n*-hexane (v:v; 7:18) eluent. The obtained product was precipitated from THF into 20-fold excess of methanol. The precipitate was filtered off and washed with methanol to collect **V1308** as a yellow solid (0.22 g, 42%). ^{1}H NMR (400 MHz, THF-d_8) δ: 7.75 (d, J = 8.3 Hz, 2H); 7.68 (d, J = 7.5 Hz, 2H); 7.23 (t, J = 7.0 Hz, 2H); 7.11 (dd, J = 8.2, 2.5 Hz, 4H); 6.87 – 6.72 (m, 18H); 6.52 (d, J = 8.8 Hz, 8H); 6.32 (d, J = 8.8 Hz, 10H); 5.56 (s, 4H); 3.78 (s, 12H); 3.66 (s, 12H) ppm. ^{13}C NMR (101 MHz, THF) δ: 160.39; 160.08; 151.35; 150.36; 146.67; 142.81; 137.14; 135.27; 133.68; 131.71; 131.52; 129.80; 128.62; 128.60; 127.65; 124.92; 121.12; 120.65; 118.22; 114.72; 113.74; 112.85; 66.50; 55.68; 55.42 ppm. Anal. calcd for $C_{89}H_{74}N_2O_8$: C 82.26; H 5.74; N 2.16; found: C 82.14; H 5.43; N 2.5. $C_{89}H_{74}N_2O_8$[M$^+$] exact mass = 1298.545, MS (MALDI-TOF) = 1299.521.

Table S1. Materials, quantities, and cost for the synthesis of **V1307**.

Chemical	Weight reagent (g/g)	Weight solvent (g/g)	Weight workup (g/g)	Price of chemical (€/kg)	Cost of chemical (€/g product)	Total cost (€/g)
4-Bromoanisole	3.98			25.2	0.10	
Formic acid	20			3.87	0.08	
Sulfuric acid	4			2.63	0.01	
Methyl methoxyacetate	0.739			304	0.22	
Magnesium	0.518			104	0.05	
Diethyl ether		200		6.92	1.38	
Ethyl acetate			150	2.85	0.43	
**2,2-bis(4-methoxyphenyl)acetaldehyde**	29.237	200	150			2.28
2,2-bis(4-methoxyphenyl)acetaldehyde	4.075			2300	9.37	
2,2’,7,7’-Tetraamino-9,9’-spirobifluorene	0.5			22500	11.25	
10-Camphorsulfonic acid	0.3			260	0.08	
Toluene		18		2.46	0.04	
Tetrahydrofuran			444	8.88	3.94	
Methanol			100	4.00	0.40	
n-Hexane			560	3.16	1.77	
Silicagel			50	64.8	3.24	
**V1307**	4.875	18	1154			30.10

V1307 estimated synthesis cost of 30.1 €/1g (37 $/1g) is indicative and calculated only for comparison.

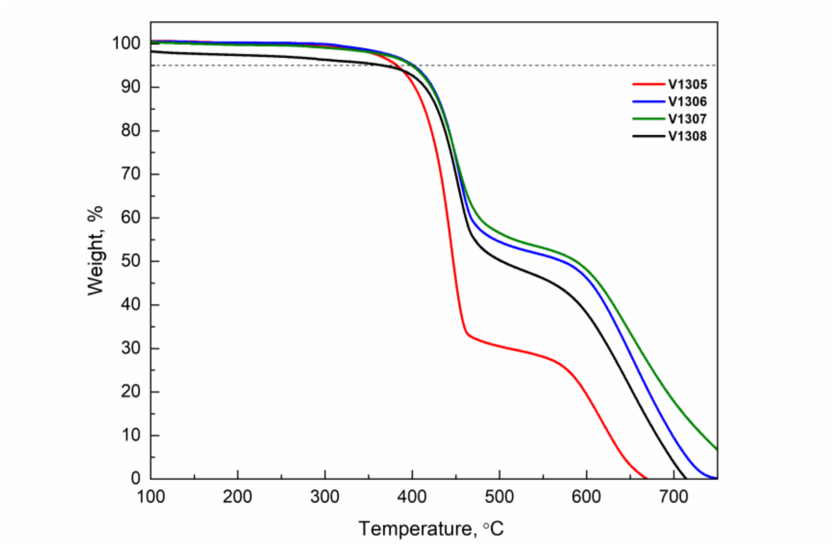

Figure S1. Thermogravimetric analysis (TGA) data (heating rate of 10 °C/min, N_2 atmosphere).

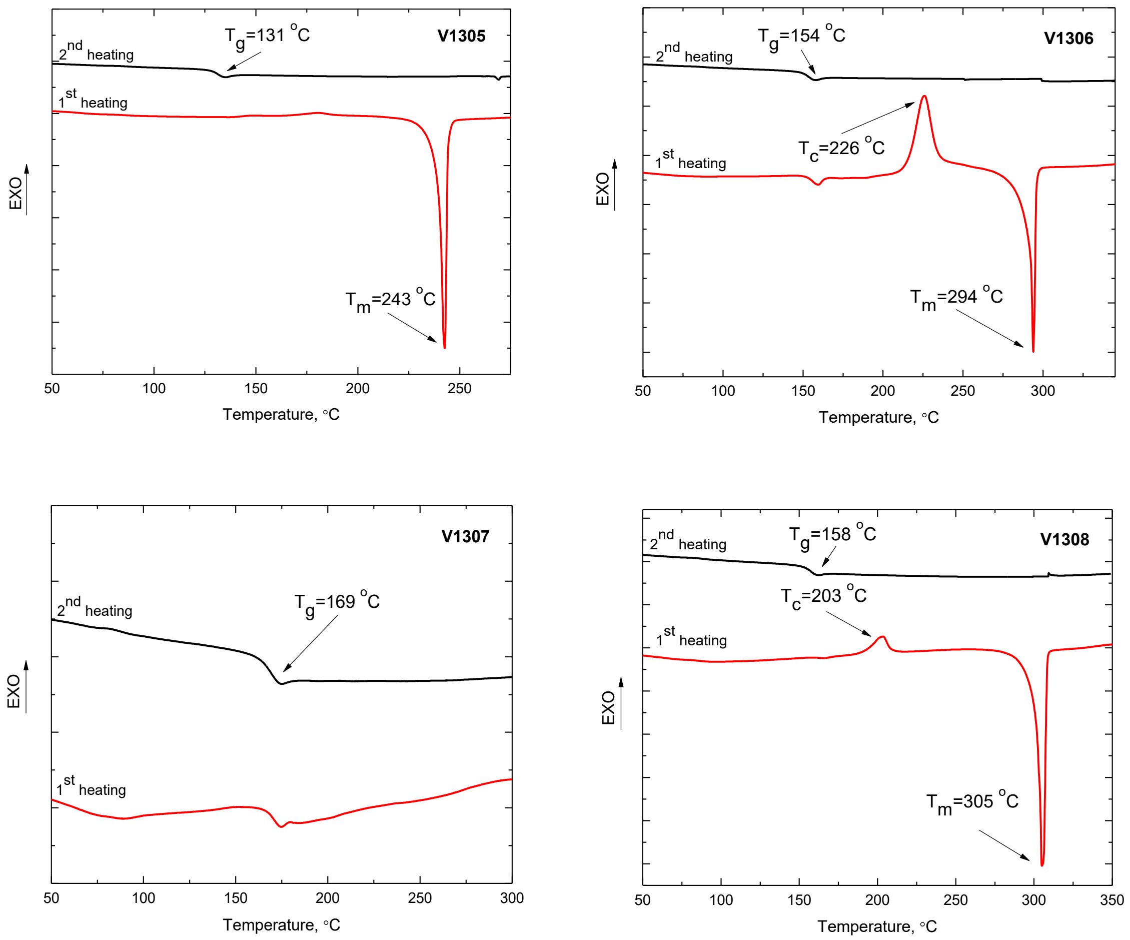

Figure S2. Differential scanning calorimetry (DSC) first and second heating curves of HTMs (scan rate 10 ºC/min, N_2 atmosphere).

V1305

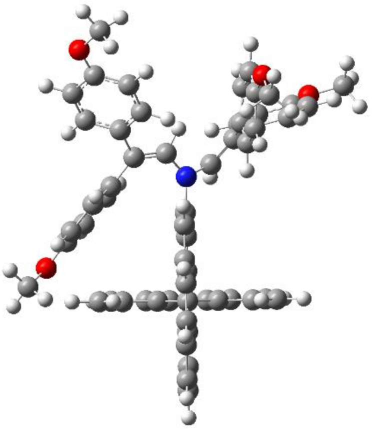

V1306

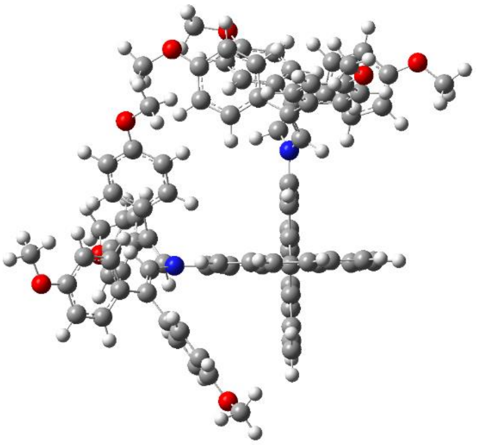

V1307

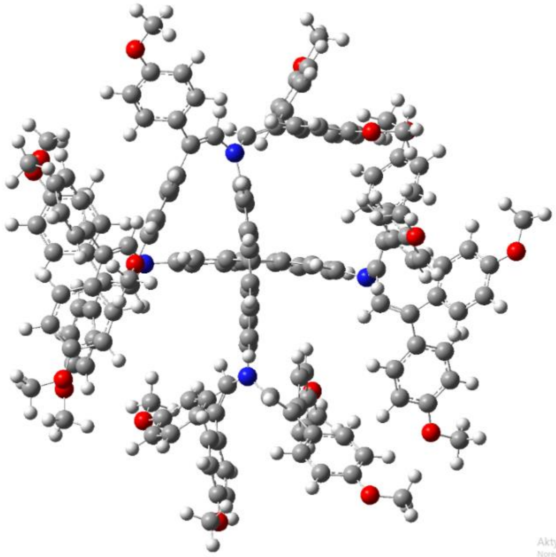

V1308

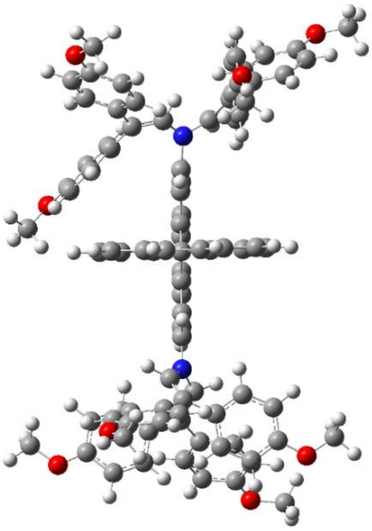

spiro-OMeTAD

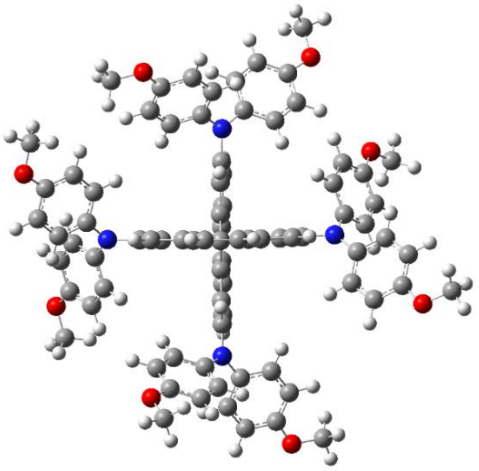

Figure S3. Optimized molecular structures of **V1305**, **V1306**, **V1307**, **V1308** (after ground state geometry optimization). Gaussian09, B3LYP/6-31G.

Semiempirical TD method (for singlets only) was used for simulation of electronic absorption spectrum. Environmental effects were not included for all simulations. **Table S1** represents the parameters of four lowest excited states: S_1, S_2, S_3, S_4. For **V1305** (one methyl-oxy-phenyl to F1), transition $S_0 \rightarrow S_1$ is partially allowed (*f*=0.24), excitation energy 3.17 eV. For **V1306** (one methyl-oxy-phenyl to F1, one methyl-oxy-phenyl to F2), transition $S_0 \rightarrow S_1$ is partially allowed (*f*=0.15), excitation energy 3.08 eV. For **V1308** (two methyl-oxy-phenyl to F1), transition $S_0 \rightarrow S_1$ is allowed (*f*=0.67), excitation energy 3.01 eV. For **V1307** (two methyl-oxy-phenyl to F1, two methyl-oxy-phenyl to F2), transition $S_0 \rightarrow S_1$ is forbidden (*f*=0.09), excitation energy 2.87 eV, otherwise, transition $S_0 \rightarrow S_2$ is allowed (*f*=0.83), excitation energy 2.95 eV. Transition between "spectroscopic" states (from ground states to excited states) ΔS and corresponding set of molecular orbitals MO with contribution coefficient *k* are presented in **Table S2**.

Table S2. Electronic absorption spectra simulated by *Gaussian09*, semiempirical TD routine (for singlets only). Population of four lowest excited states: S_1, S_2, S_3, S_4. Transition energy ΔE and corresponding oscillator strength *f*.

	$S_0 \rightarrow S_1$		$S_0 \rightarrow S_2$		$S_0 \rightarrow S_3$		$S_0 \rightarrow S_4$	
	ΔE, eV	*f*	ΔE, eV	*f*	ΔE, eV	*f*	ΔE, eV	*f*
**V1305**	3.17	0.240	3.23	0.343	3.40	0.000	3.68	0.038
**V1306**	3.08	0.151	3.09	0.216	3.18	0.003	3.31	0.634
**V1307**	2.87	0.088	2.95	0.829	2.98	0.016	3.02	0.234
**V1308**	3.01	0.688	3.07	0.171	3.10	0.008	3.17	0.482

Table S3. Electronic excitations of **V1305**, **V1306**, **V1307**, **V1308**, simulated by *Gaussian09*, semiempirical TD routine (for singlets only). Population of four lowest excited states: S_1, S_2, S_3, S_4. Transition between “spectroscopic” states (from ground states to excited states) ΔS and corresponding set of molecular orbitals MO with contribution coefficient *k* (contribution of the respective excitation to the configurational interaction wavefunction.

	ΔS	MO	*k*	ΔS	MO	*k*
**V1305**	$S_0 \to S_1$	HOMO→LUMO	0.35909	$S_0 \to S_2$	HOMO→LUMO	0.60136
		HOMO→LUMO+1	0.60261		HOMO→LUMO+1	-0.35932
	$S_0 \to S_3$	HOMO→LUMO+2	0.70360	$S_0 \to S_4$	HOMO→LUMO+3	0.67342
**V1306**	$S_0 \to S_1$	HOMO-1→LUMO	0.67742	$S_0 \to S_2$	HOMO→LUMO+1	0.69335
	$S_0 \to S_3$	HOMO→LUMO	0.68687	$S_0 \to S_4$	HOMO→LUMO+2	0.68996
**V1307**	$S_0 \to S_1$	HOMO-2→LUMO	0.38690	$S_0 \to S_2$	HOMO→LUMO+1	0.65904
		HOMO-1→LUMO	0.52268			
		HOMO→LUMO	-0.20461			
	$S_0 \to S_3$	HOMO→LUMO	0.66214	$S_0 \to S_4$	HOMO-1→LUMO+1	-0.36720
					HOMO→LUMO+2	0.53003
**V1308**	$S_0 \to S_1$	HOMO→LUMO	0.68773	$S_0 \to S_2$	HOMO→LUMO+1	0.47611
					HOMO→LUMO+2	-0.34864
	$S_0 \to S_3$	HOMO→LUMO+1	0.44499	$S_0 \to S_4$	HOMO→LUMO+3	0.64929
		HOMO→LUMO+2	0.53996			

Distributions of electron density for the HOMO and HOMO-1 as well as the LUMO and LUMO+1 for **V1305**, **V1306**, **V1307**, **V1308**, structures are presented in **Figures S4-S5**. Pure CT charge redistribution behaviour (between core F1>C<F2 and substituents) was established for **V1307** HOMO-LUMO transition. Three types of charge redistribution were established.

Firstly, charge redistribution from methoxyphenyl to F1 core (**V1305**, HOMO→LUMO transition; **V1306**, HOMO→LUMO+1 transition; **V1307**, HOMO→LUMO+1 transition; **V1308**, HOMO→LUMO transition) is typical for all compounds. Methoxyphenyl fragment plays the role of charge donor, and charge acceptor is fluorene, when certain substituent is related in C4 position.

Secondly, presence of additional substituents rapidly changes the behaviour of excitation. For **V1306** (one methoxyphenyl to F1, one methoxyphenyl to F2), HOMO→LUMO transition represents charge redistribution between methxyphenyl fragments, related to the different fluorene cores (each fluorene core is involved insignificantly). Different methoxyphenyl fragments play role of charge donor and charge acceptor, respectively. For mentioned motion, two perpendicular fluorenes (F1<C>F2) play role of bridge. The same situation is repeated for **V1307** (two methoxyphenyl to F1, two methxyphenyl to F2), HOMO→LUMO transition.

Thirdly, presence of two methoxyphenyl substituents at 4 and 11 position of the same F1 core allows charge redistribution between bottom substituents and top substituents through fluorene F1 bridge – see **V1307**, HOMO-1→LUMO transition.

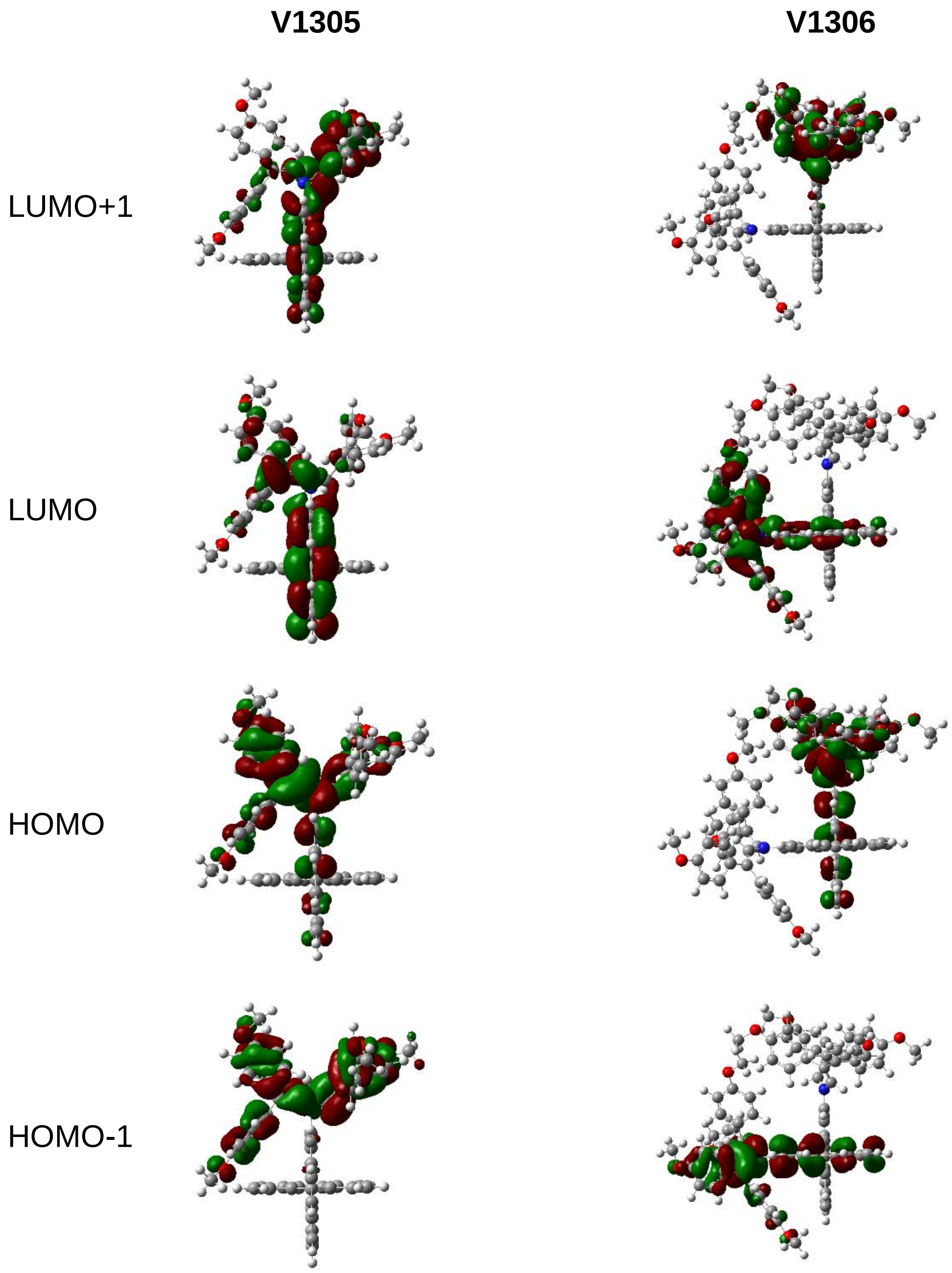

Figure S4. V1305 and **V1306** distributions of electron density for the HOMO-1, HOMO, LUMO and LUMO+1.

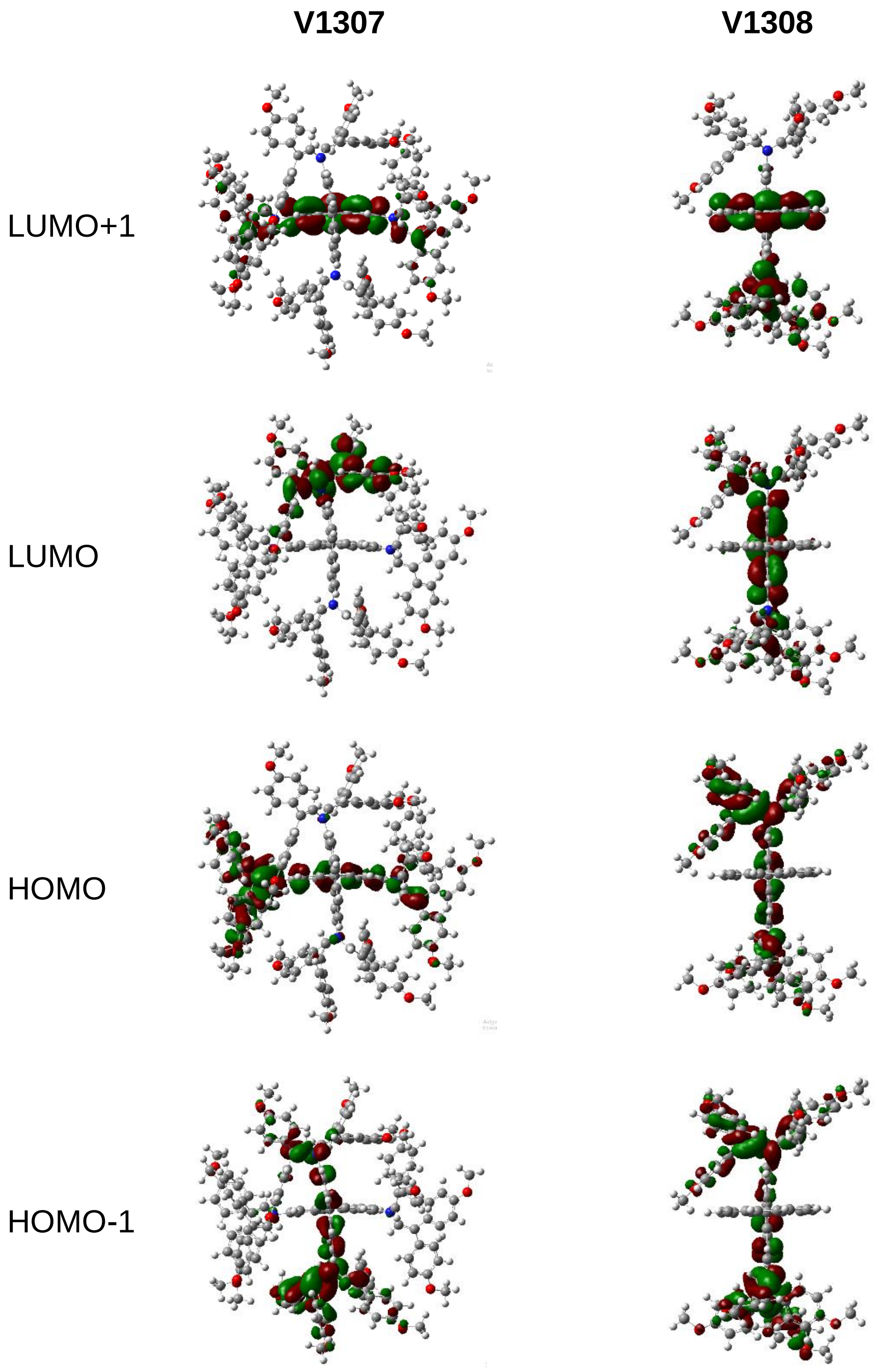

Figure S5. V1307 and **V1308** distributions of electron density for the HOMO-1, HOMO, LUMO and LUMO+1.

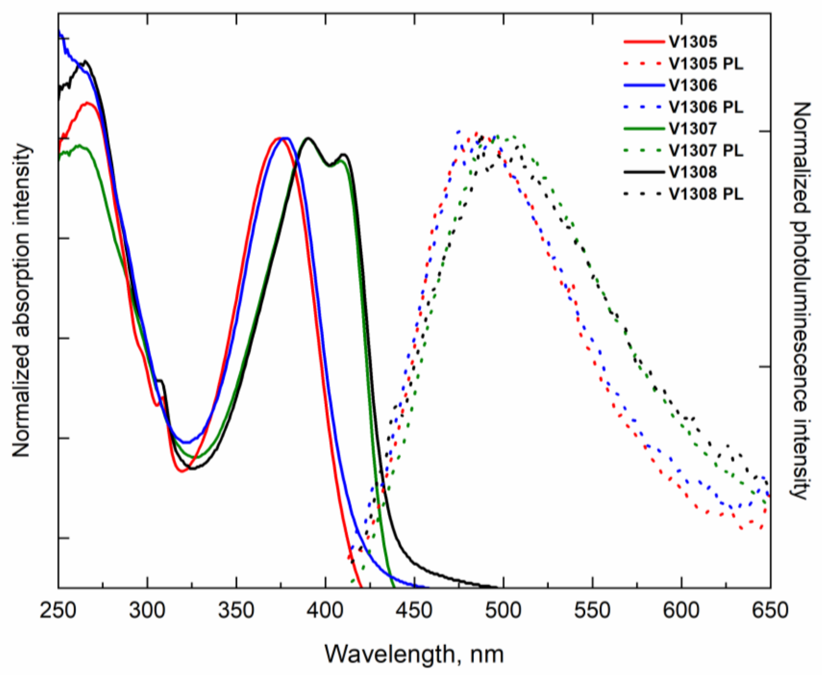

Figure S6. UV−Vis absorption (solid line) and photoluminescence (dashed line) spectra of thin films V-series HTMs.

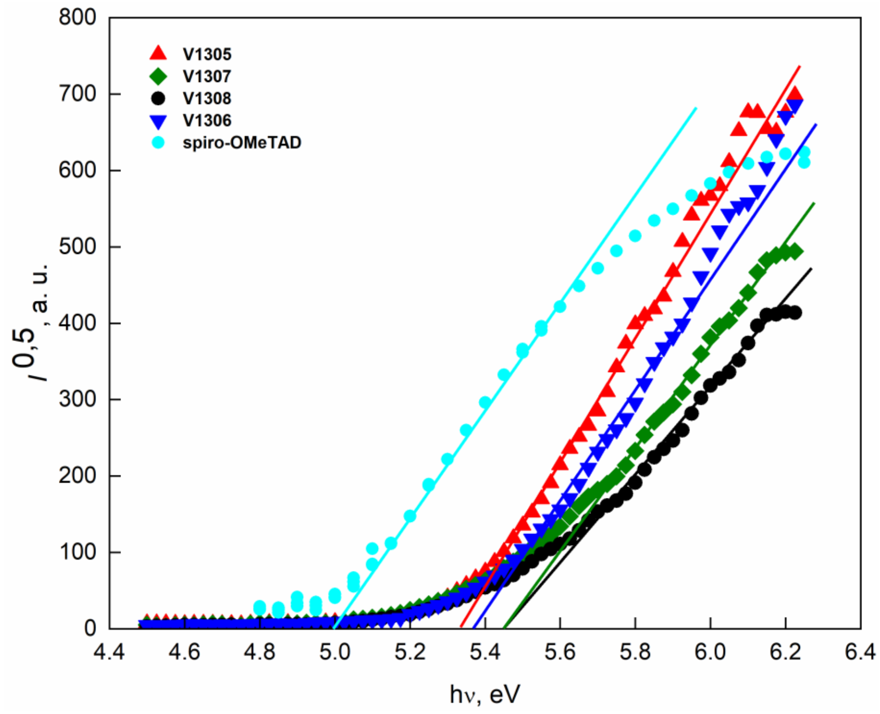

Figure S7. Photoemission in air spectra of the charge transporting layers **V1305**, **V1306**, **V1307** and **V1308**.

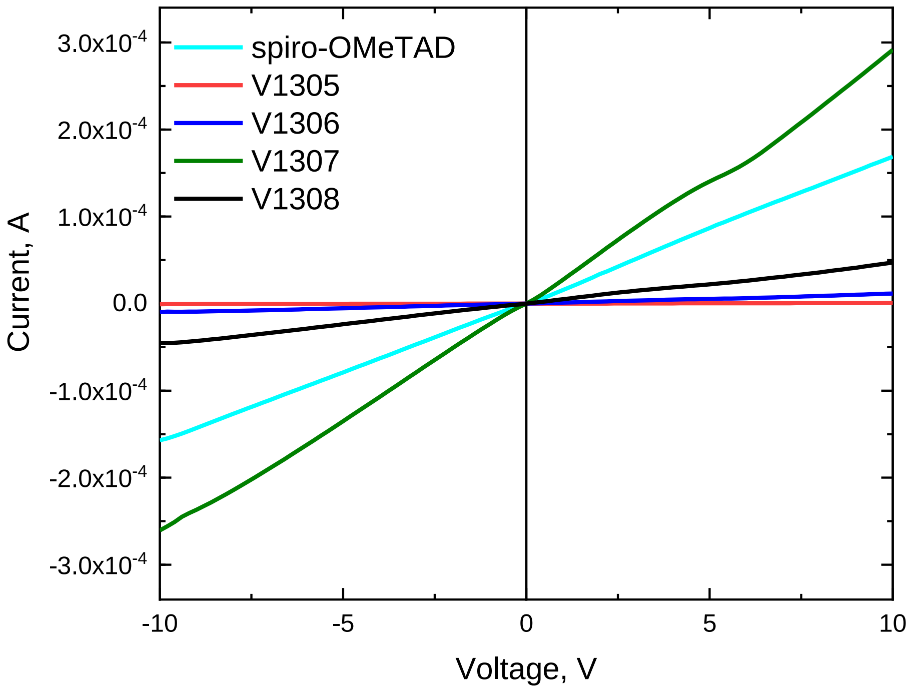

Figure S8. The current-voltage measurements for calculating the lateral conductivity of **V1305**, **V1306**, **V1307**, **V1308** and spiro-OMeTAD measured on OFET substrates.

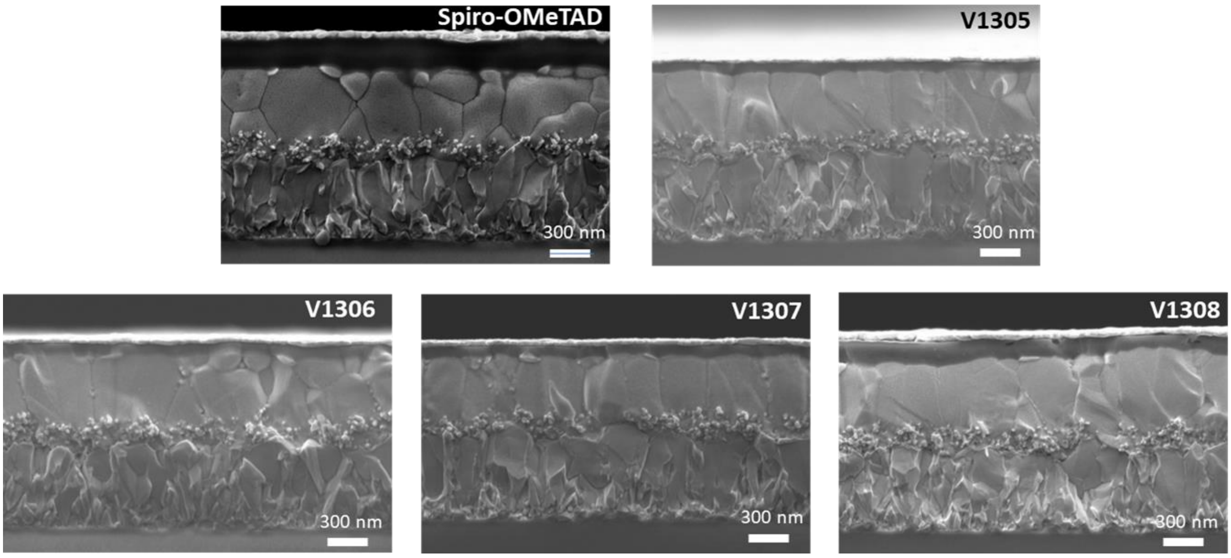

Figure S9. Cross-sectional SEM images of complete solar cells fabricated with spiro-OMeTAD, **V1305**, **V1306**, **V1307** and **V1308**, respectively.

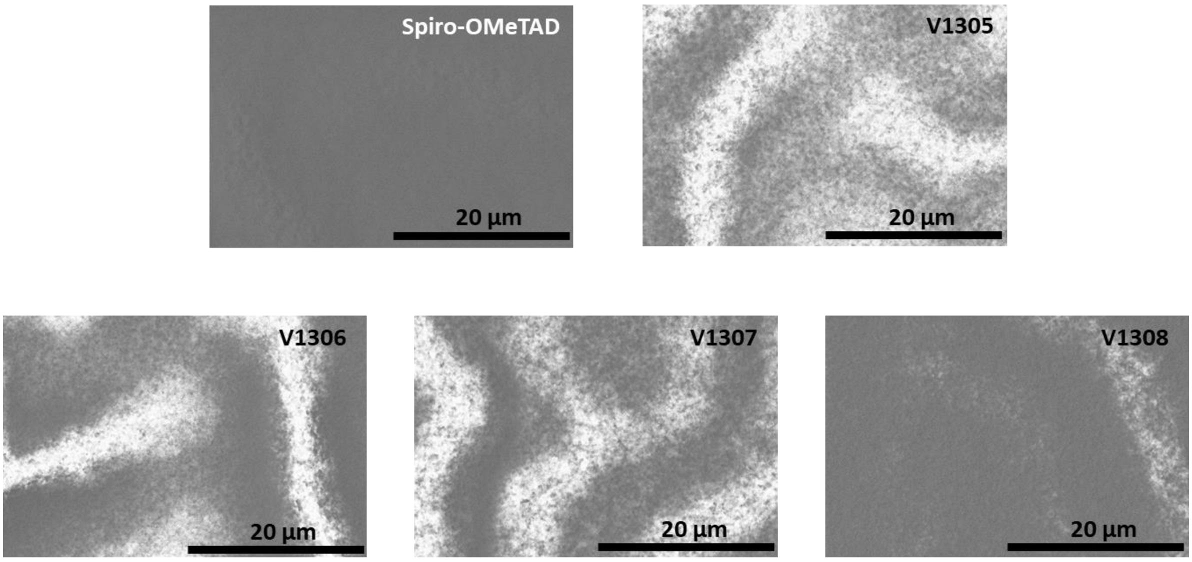

Figure S10. Top-view SEM images of spiro-OMeTAD, **V1305**, **V1306**, **V1307** and **V1308** thin films, respectively, deposited on top of perovskite.

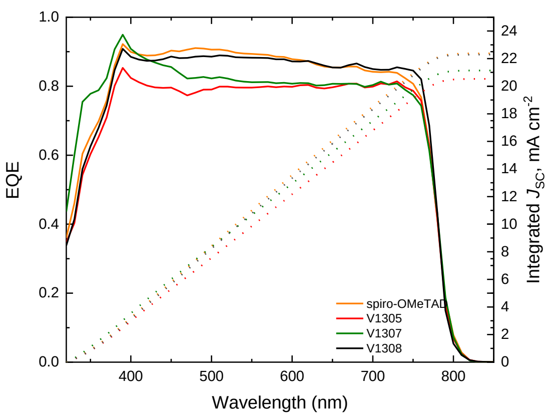

Figure S11. External quantum efficiency (EQE) of devices containing **spiro-OMeTAD**, **V1305**, **V1306**, **V1307** and **V1308**, respectively.

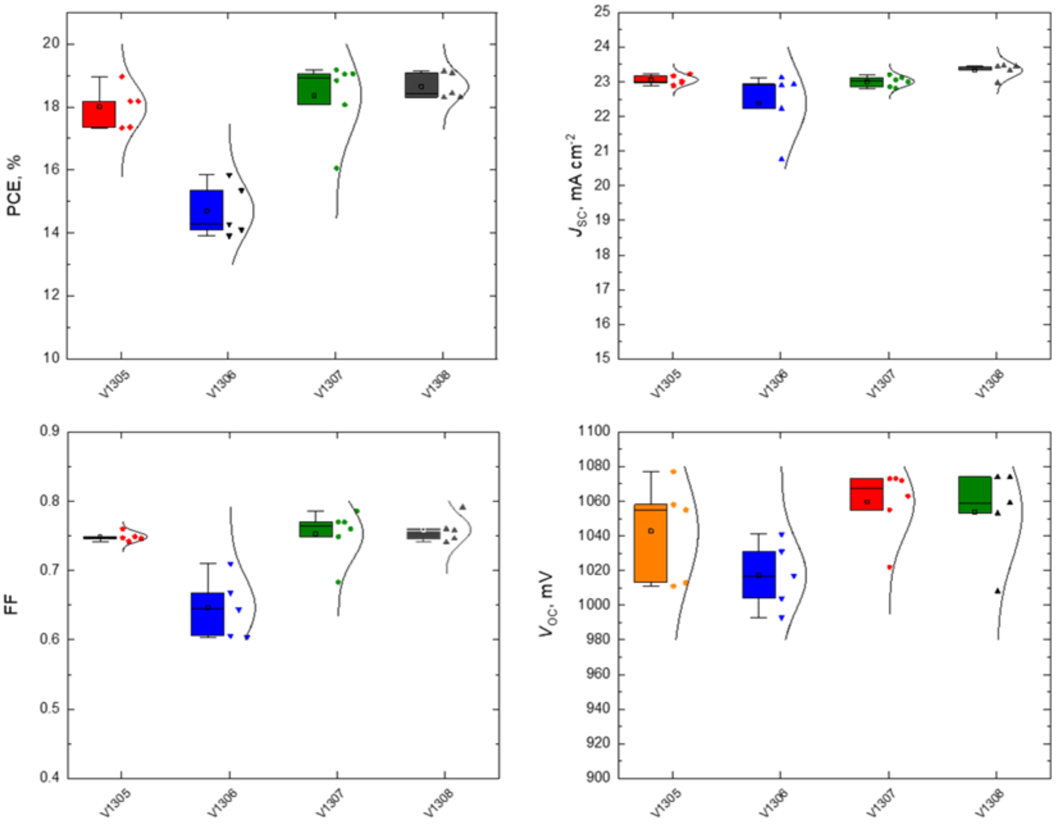

Figure S12. Statistics of solar cells fabricated with **V1305**, **V1306**, **V1307** and **V1308** HTMs. a) Power conversion efficiency, b) short-circuit current density, c) fill factor and d) open-circuit voltage. All values were extracted from the corresponding *J-V* curves.

Table S4. Average PV values extracted from the analysis of the *J-V* curves from the statistical study.

ID	V_{OC} *(mV)*	J_{SC} (mA cm^{-2})	FF	PCE (%)
**V1305**	1043±29	23.05±0.14	0.75±0.01	18.0±0.7
**V1306**	1017±19	22.39±0.97	0.65±0.04	15.8±0.9
**V1307**	1067±8	23.01±0.17	0.77±0.01	18.8±0.5
**V1308**	1054±27	23.33±0.20	0.76±0.02	18.7±0.4

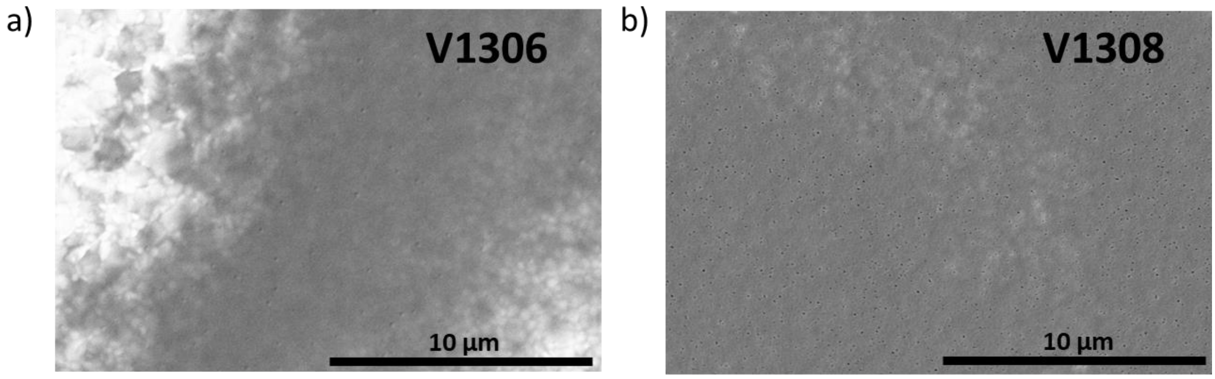

Figure S13. Top-view SEM images of **V1306** and **V1308** thin films, respectively, deposited on top of perovskite.

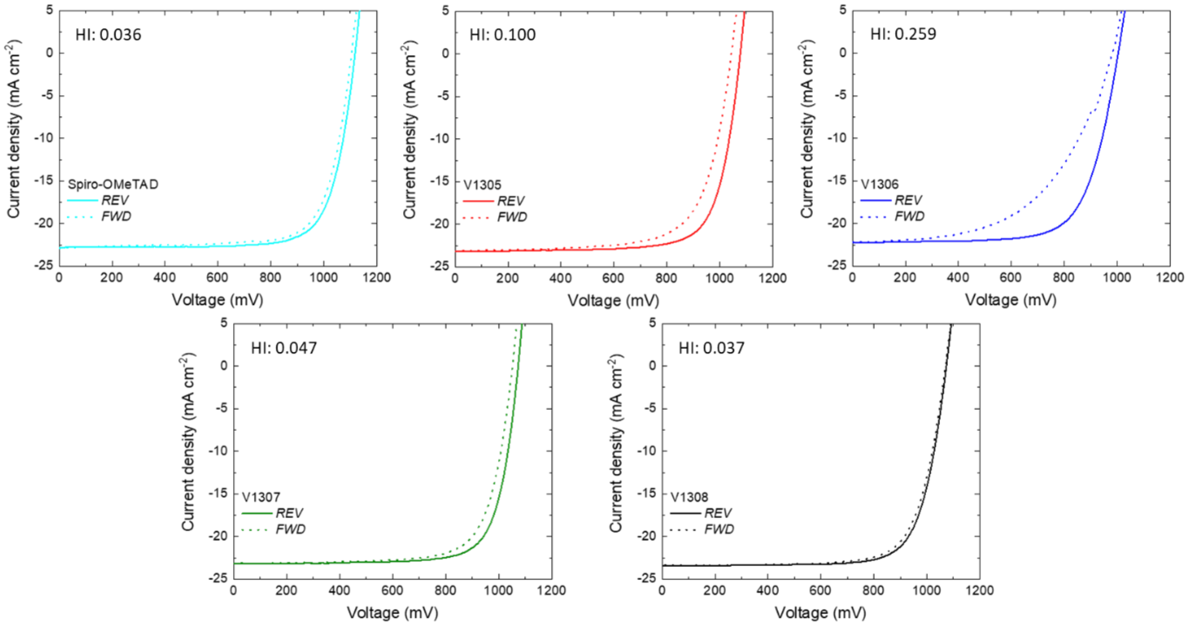

Figure S14. Hysteresis behaviour of the perovskite solar cells containing spiro-OMeTAD, **V1305**, **V1306**, **V1307** and **V1308**, respectively. The *J*-*V* curves were measured under 1 sun intensity illumination, scanned from open-circuit to short-circuit followed by a scan from short-circuit to open-circuit conditions, with a scan rate of 50 mV s^{-1} and 10 mV voltage step. HI: hysteresis index.

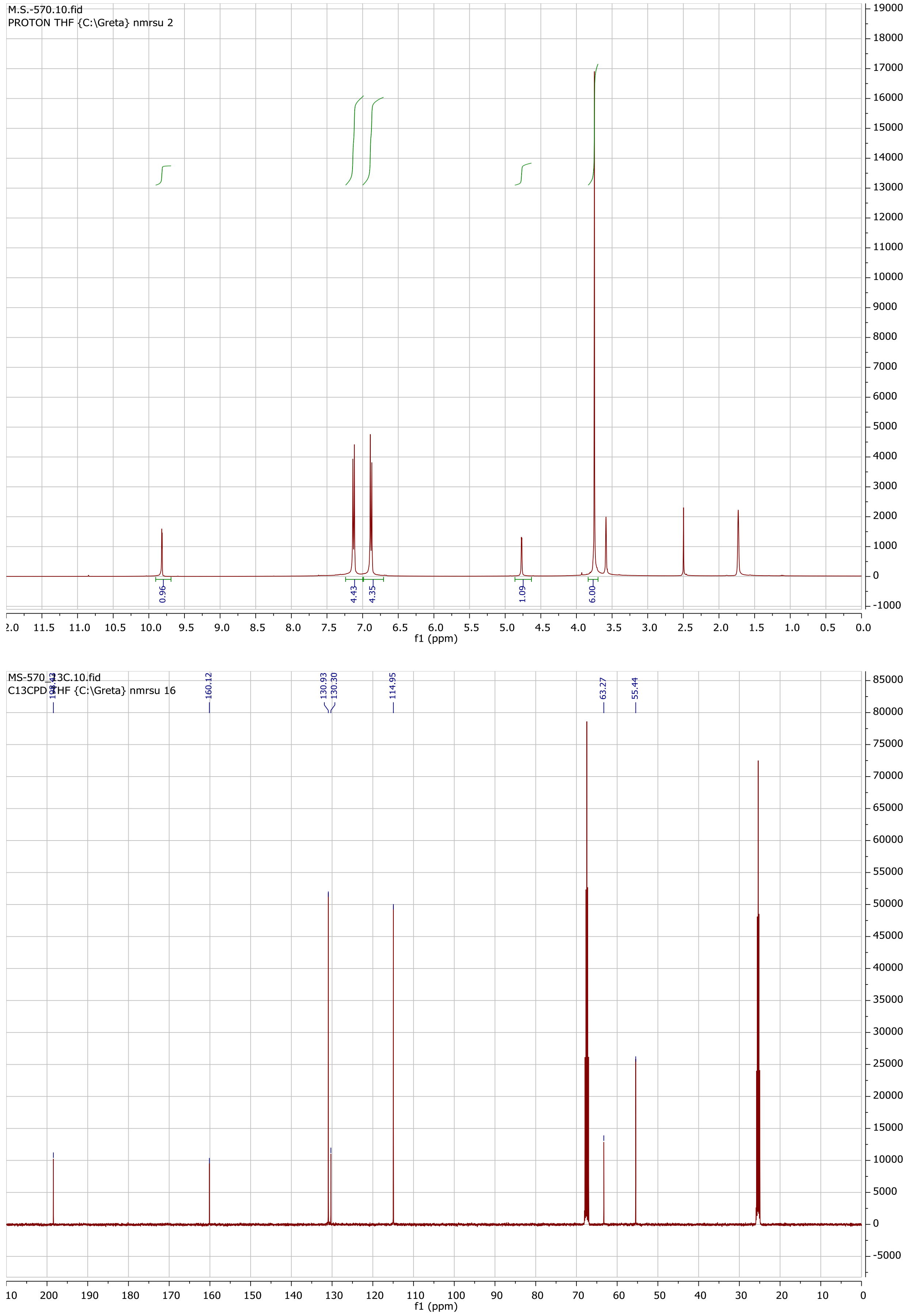

Figure S15. ^{1}H and ^{13}C NMRs of 2,2-bis(4-methoxyphenyl)acetaldehyde.

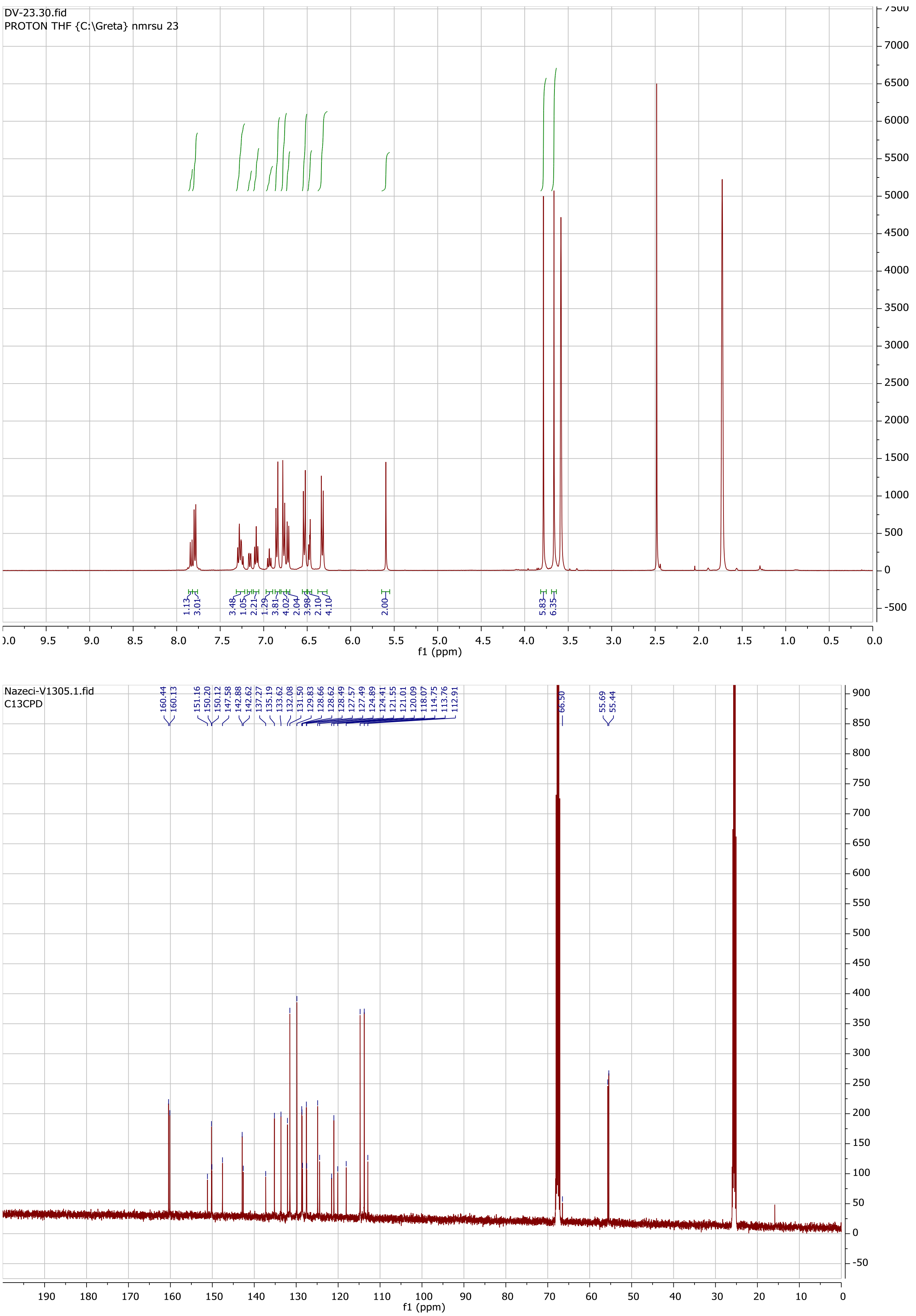

Figure S16. ^{1}H and ^{13}C NMRs of **V1305**.

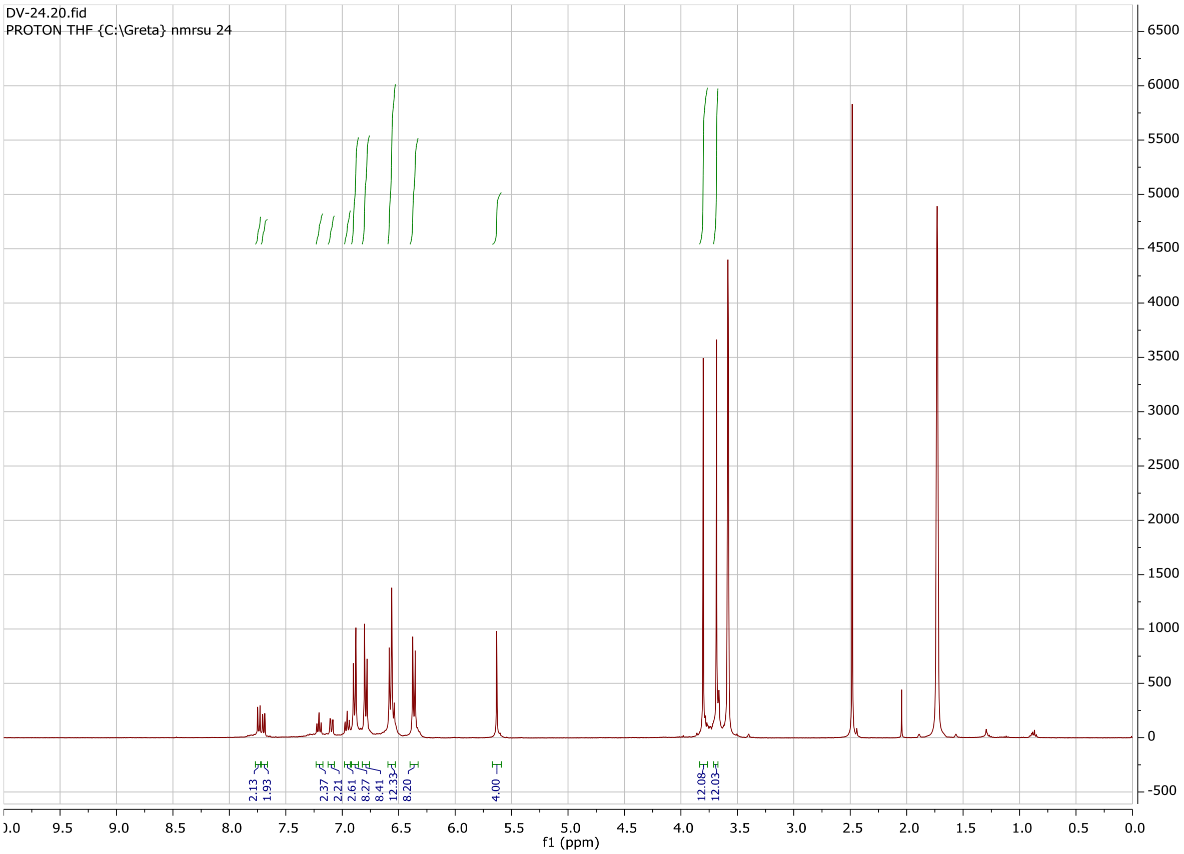

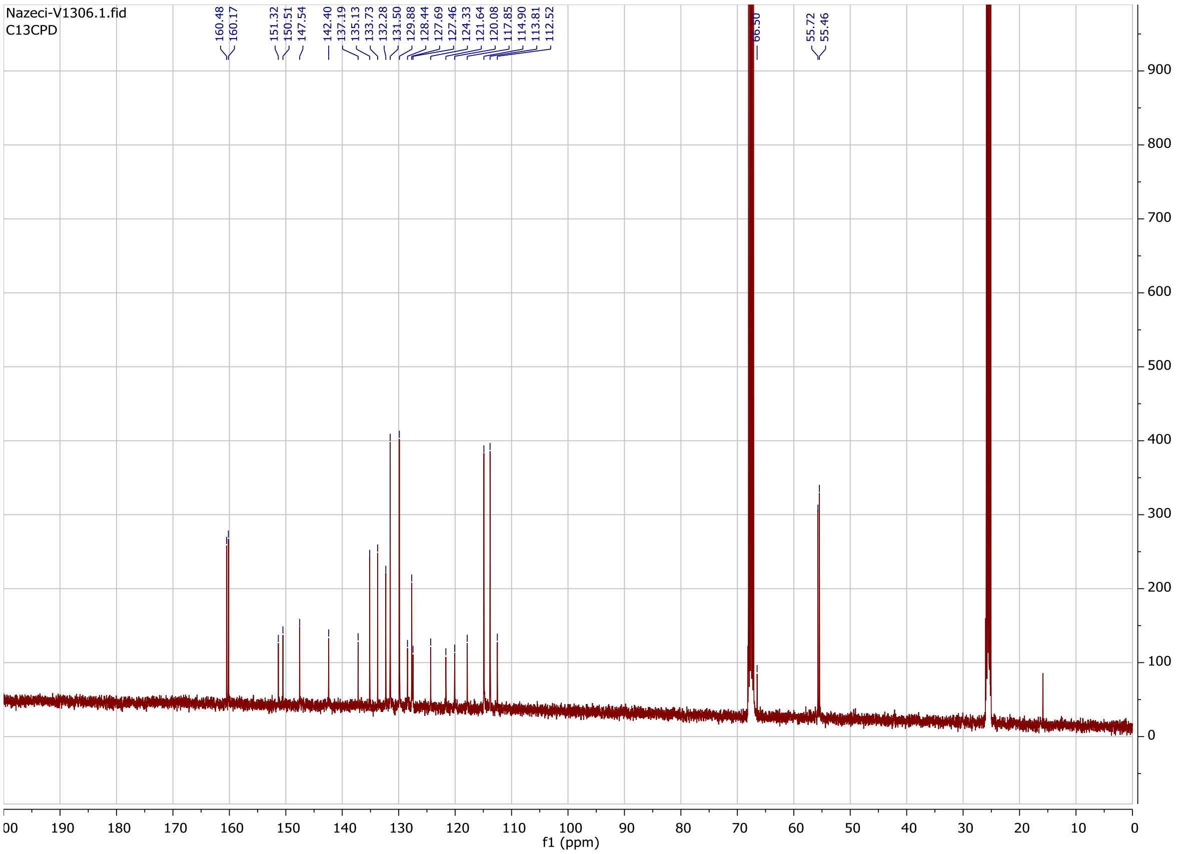

Figure S17. ^{1}H and ^{13}C NMRs of **V1306**.

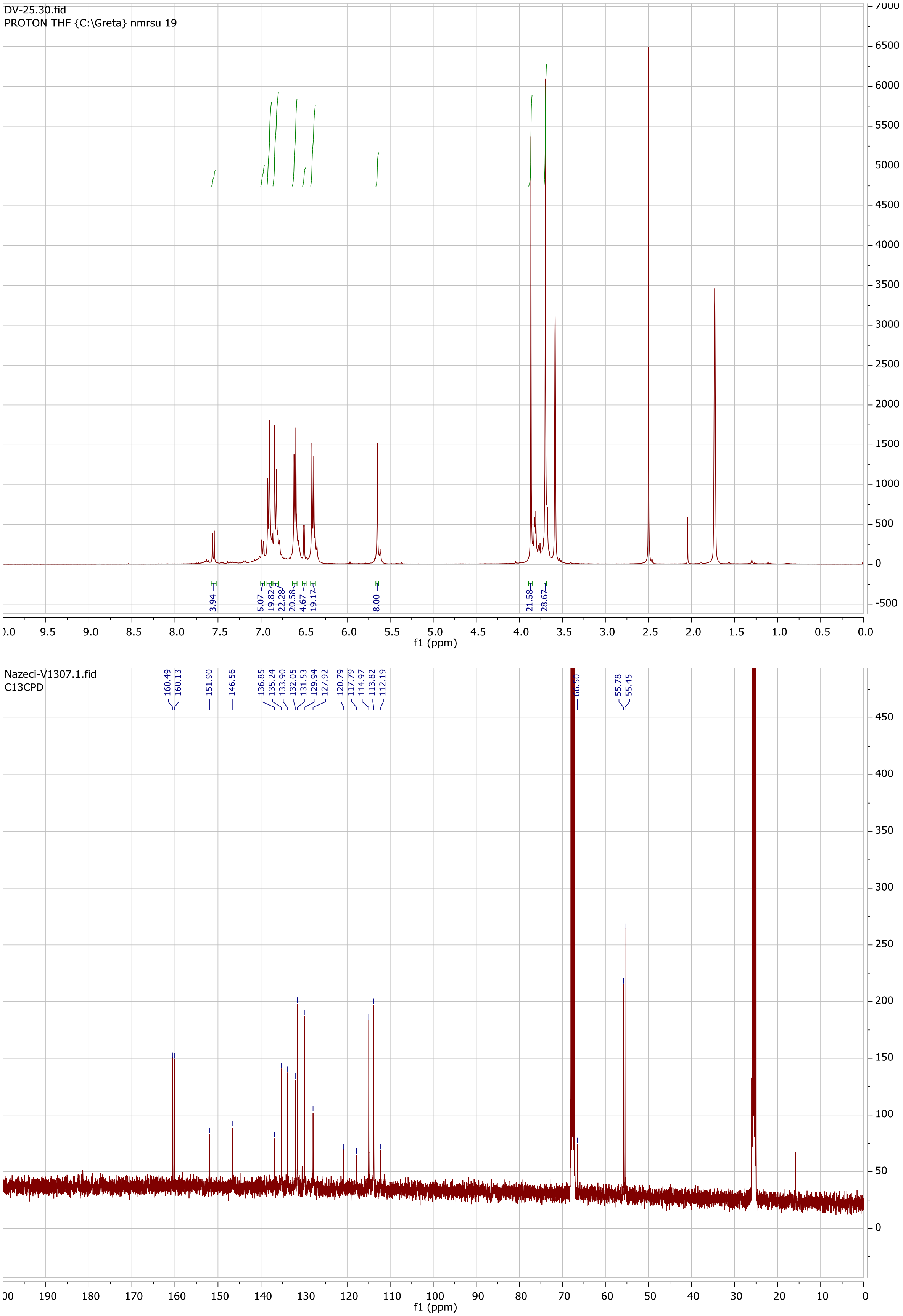

Figure S18. ^{1}H and ^{13}C NMRs of **V1307**.

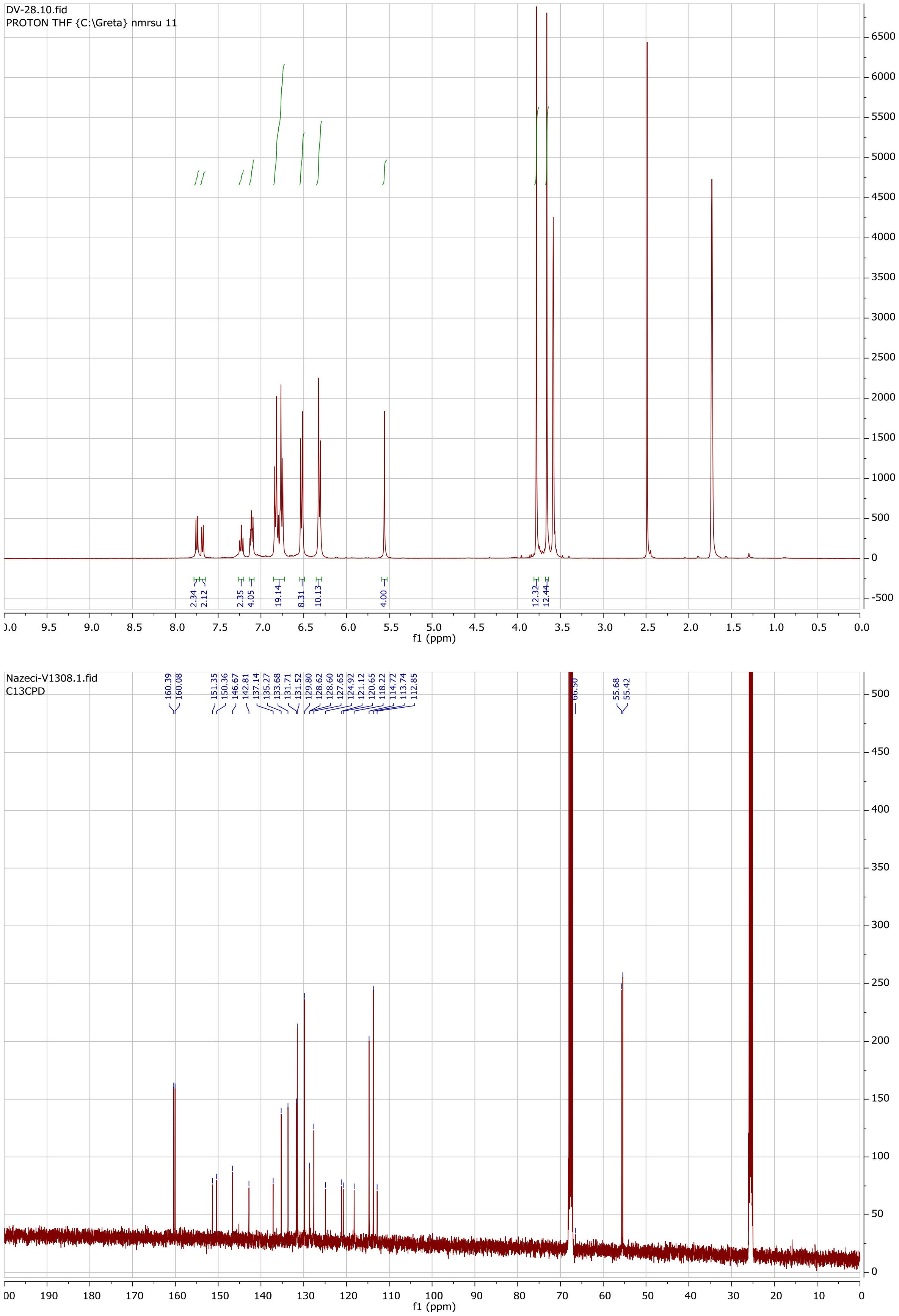

Figure S19. ^{1}H and ^{13}C NMRs of **V1308**.

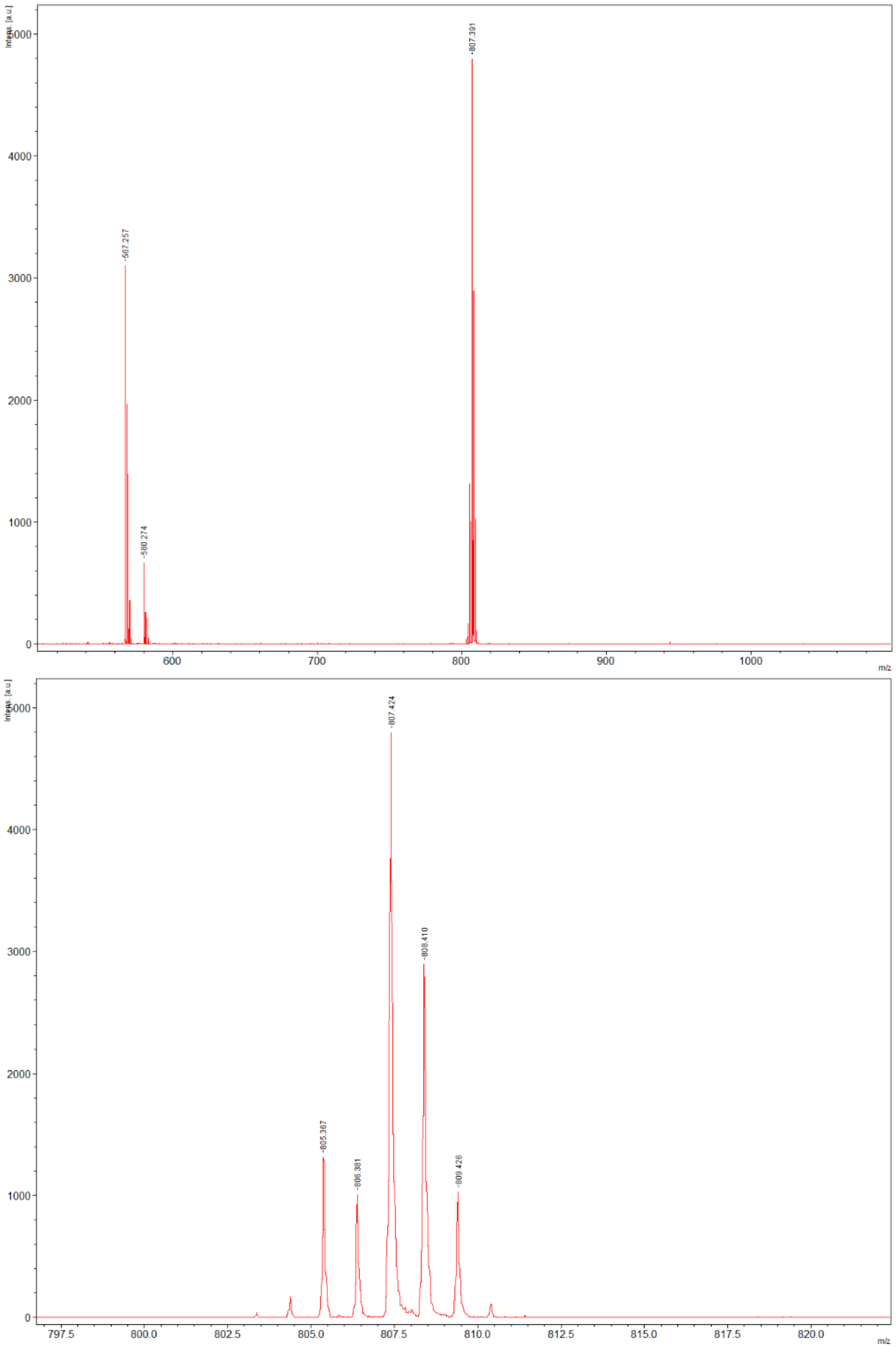

Figure S20. MALDI-TOF-MS spectra in wide and narrow mass ranges of **V1305**.

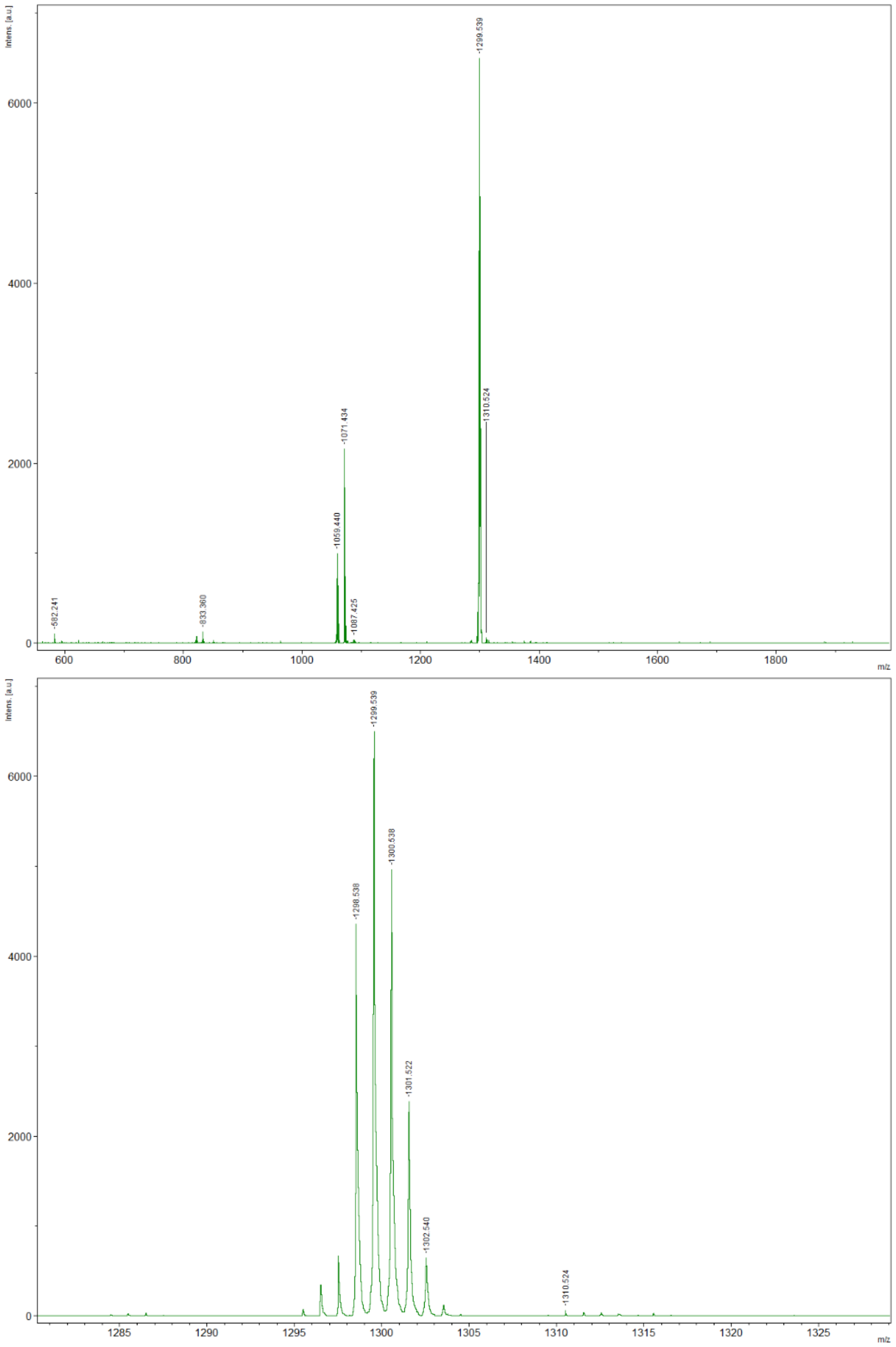

Figure S21. MALDI-TOF-MS spectra in wide and narrow mass ranges of **V1306**.

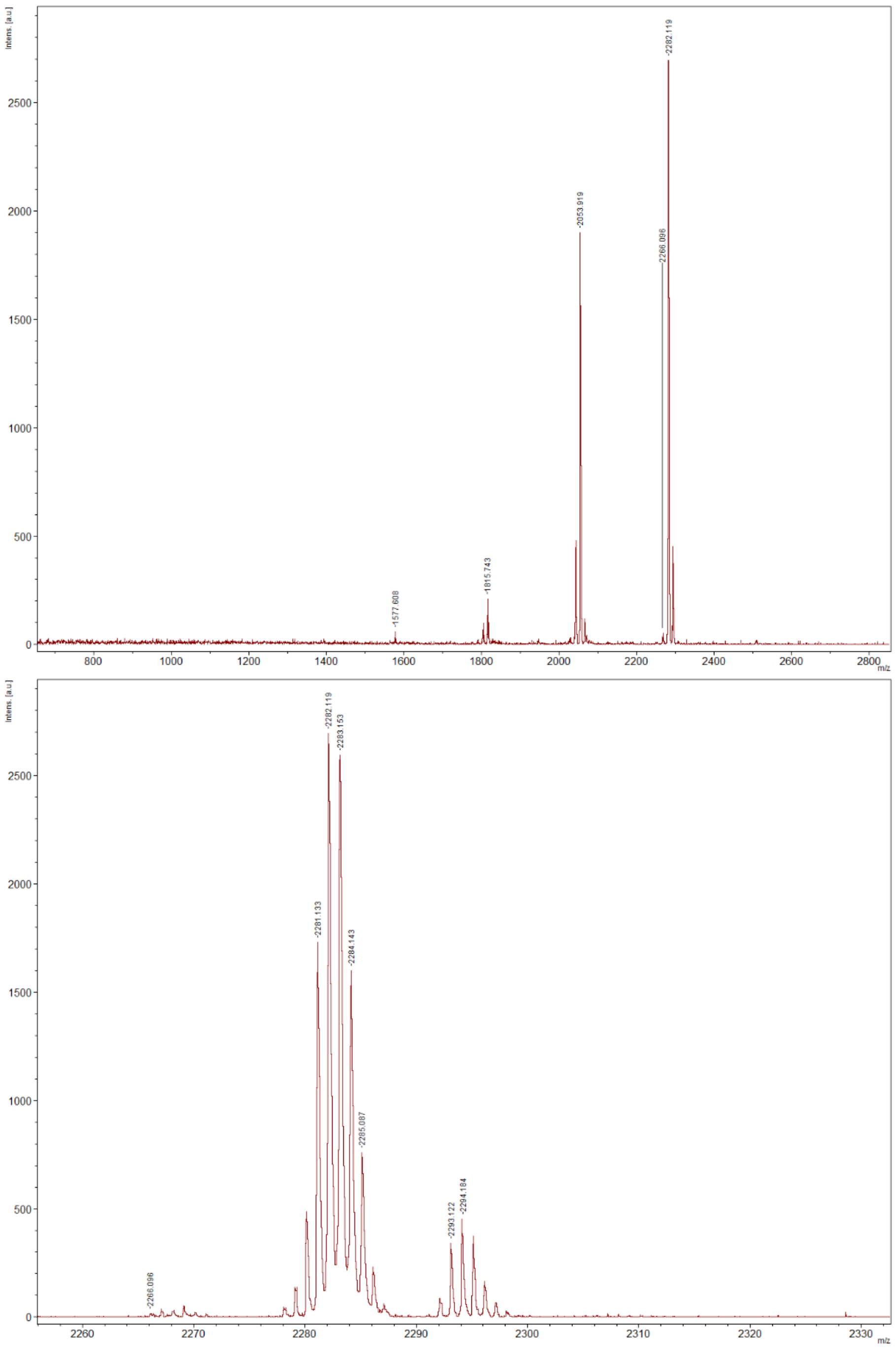

Figure S22. MALDI-TOF-MS spectra in wide and narrow mass ranges of **V1307**.

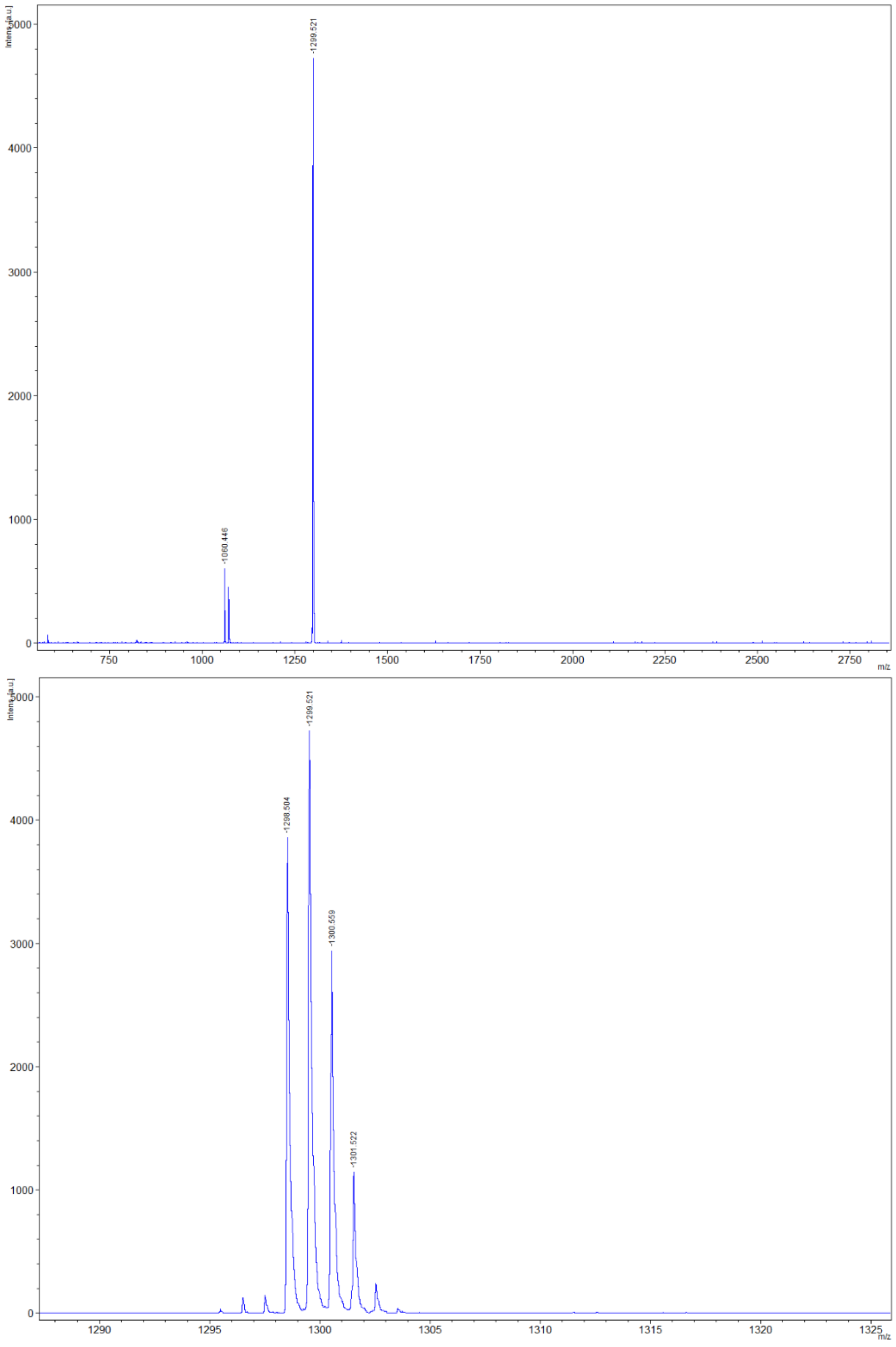

Figure S23. MALDI-TOF-MS spectra in wide and narrow mass ranges of **V1308**.

1. P. Wen, Y. Kim, H. Chun, S. Y. Yang, M. H. Lee, Mater. Chem. Phys. 2013, 139, 923.
